# Supplementary material for: A fish cartel for Africa
Source: Nat Commun. 2023 Nov 13;14:7124. doi: 10.1038/s41467-023-42886-z (PMC10643414; doi:10.1038/s41467-023-42886-z)
Supplement: Supplementary file 1 — Supplementary Information [file 41467_2023_42886_MOESM1_ESM.pdf]

Supplementary Information for

A fish cartel for Africa

Gabriel Englander, Christopher Costello

Correspondence to: aenglander@worldbank.org

This PDF file includes:

Figures S1 to S27
Tables S1 to S11

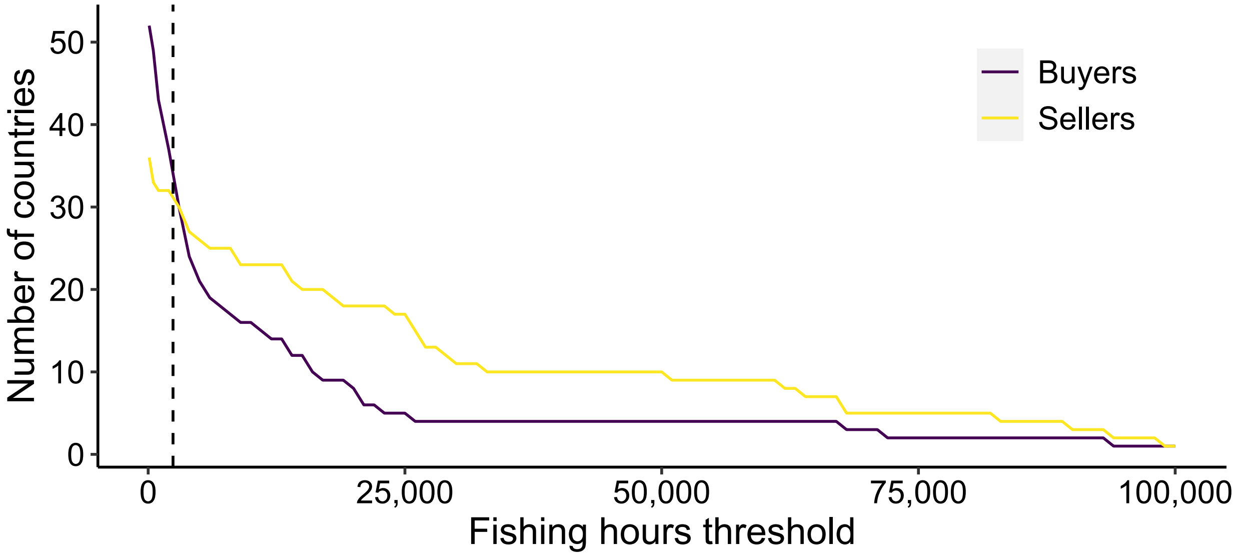

Figure S1: **Number of buying and selling countries as a function of the fishing hours threshold.** The y-axis displays the number of selling and buying countries as the fishing hours threshold (x-axis) changes. The vertical dashed line is the largest fishing hours threshold that would include in our model all selling countries for whom we observe an access agreement between 2016 and 2020. We use the same threshold to identify buying countries. This threshold is 2,420 fishing hours, which yields 32 African countries that sell access to their waters and 33 countries that purchase access.

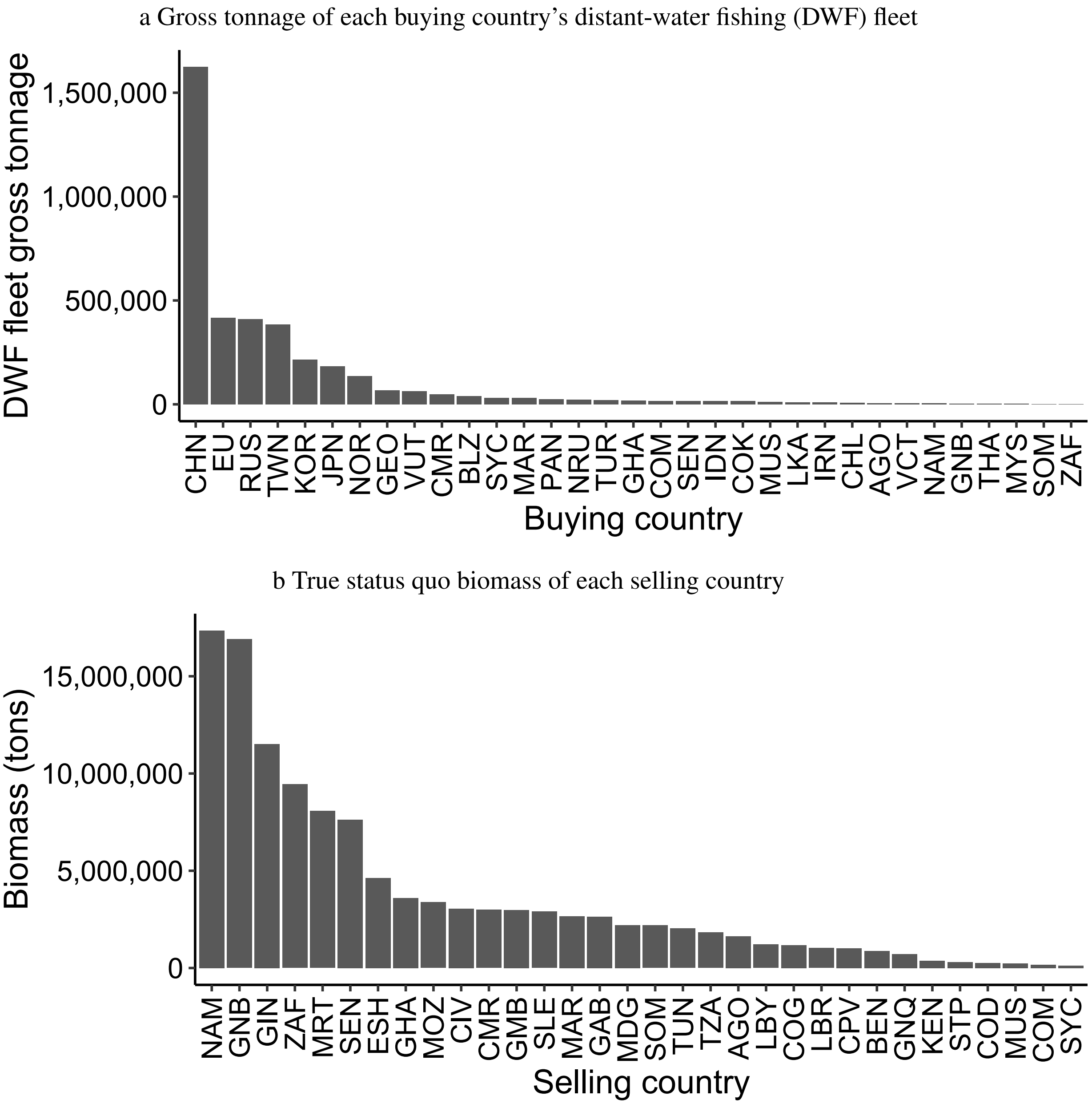

Figure S2: **(a) Gross tonnage of each buying country's distant-water fishing (DWF) fleet and (b) True status quo biomass of each selling country.** The x-axes display the ISO3 code of each country. We model the European Union (EU) as a single country because the EU purchases fishing access as a bloc.

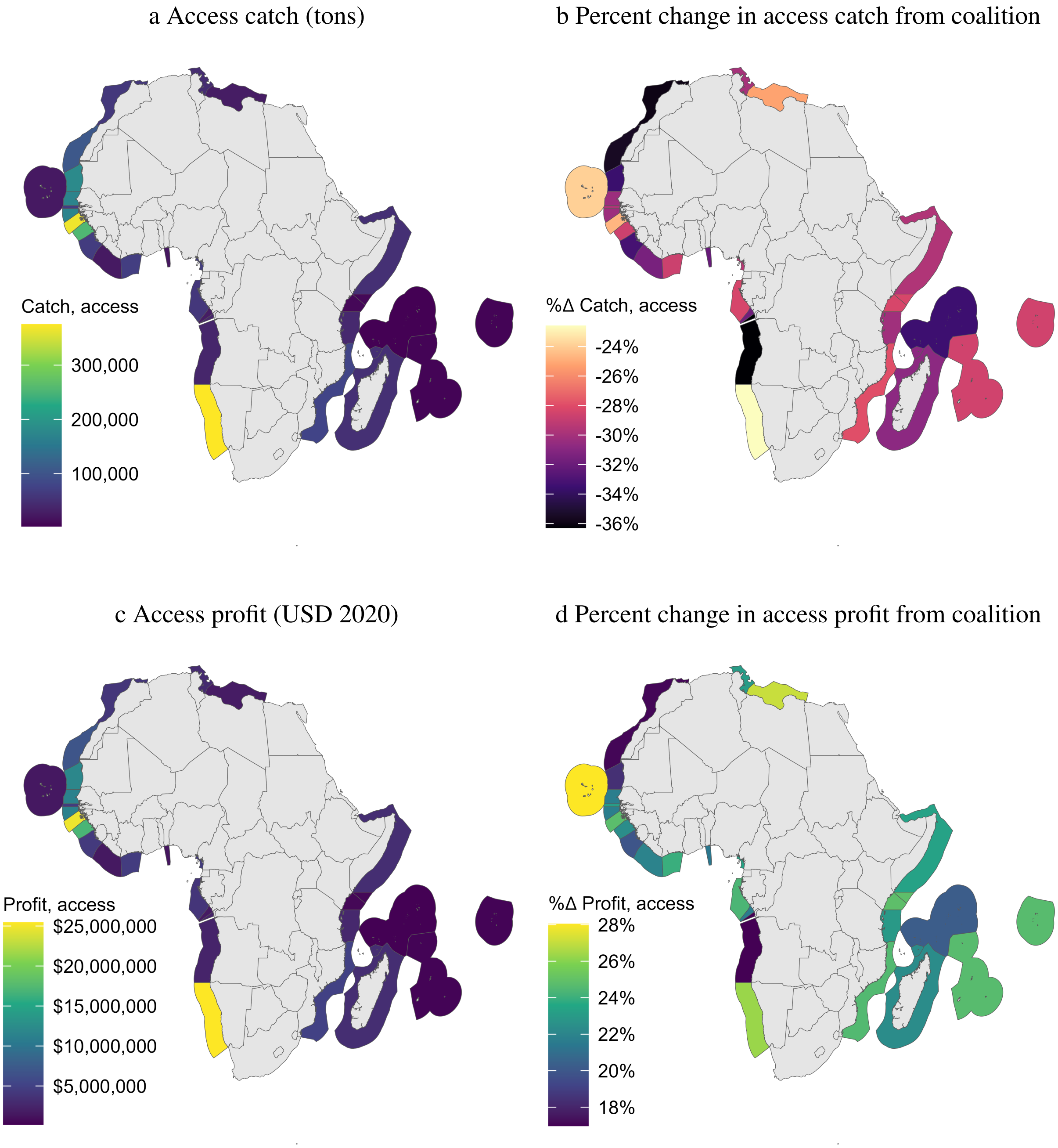

Figure S3: **Effect of Africa Coalition on access catch and profit by selling country when fishing hours threshold is twice the baseline value.** (a) Access catch and (c) profit are status quo values while (b) access catch and (d) profit display the percent changes under the coalition scenario relative to the status quo values. The baseline fishing hours threshold is 2,420 (Methods). As a robustness check, we use a fishing hours threshold of 4,840 to identify selling and buying countries, and then we repeat our analysis with these selling and buying countries.

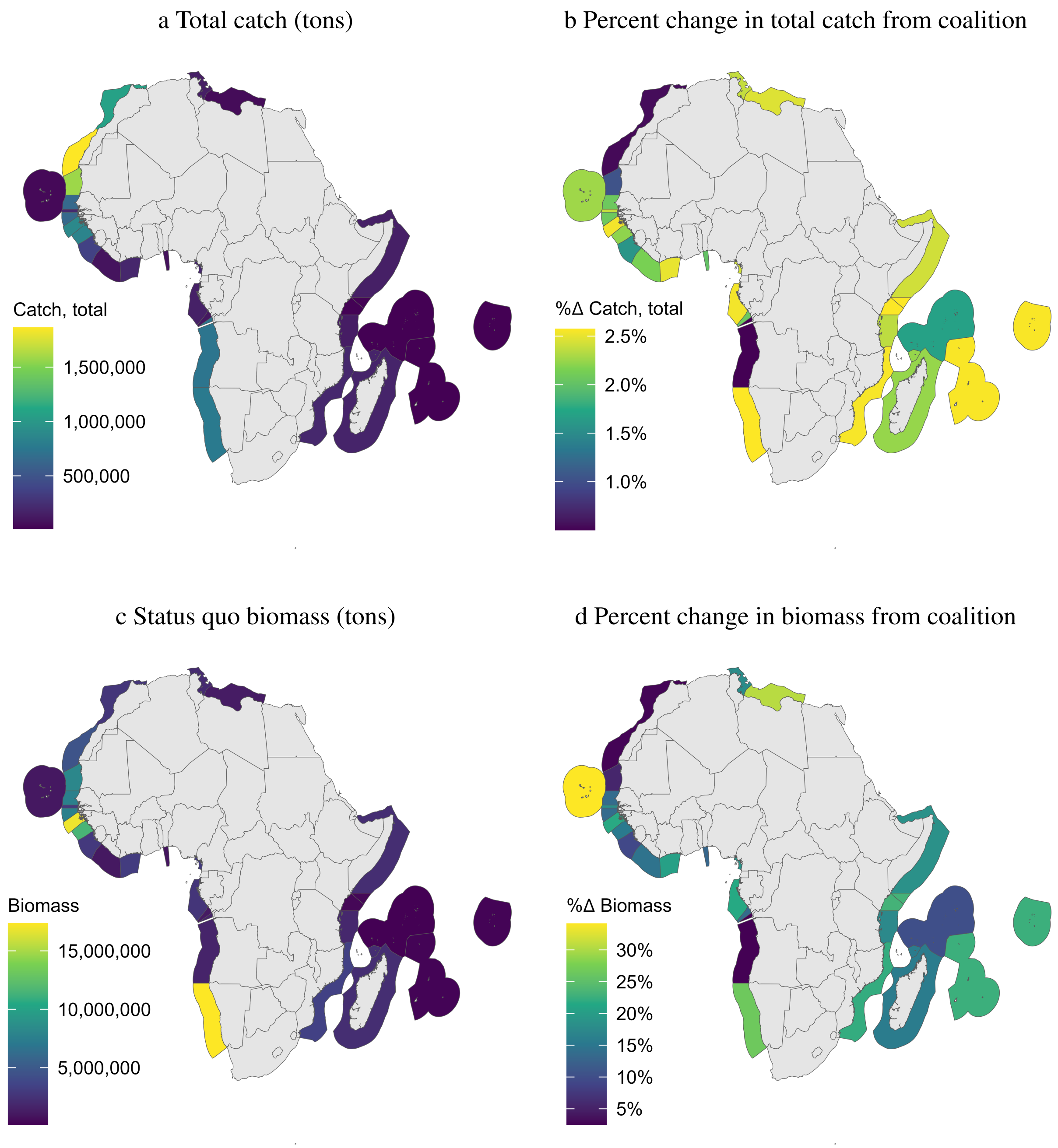

Figure S4: **Effect of Africa Coalition on total catch and biomass by selling country when fishing hours threshold is twice the baseline value.** (a) Total catch and (c) biomass are status quo values while (b) total catch and (d) biomass display the percent changes under the coalition scenario relative to the status quo values. The baseline fishing hours threshold is 2,420 (Methods). As a robustness check, we use a fishing hours threshold of 4,840 to identify selling and buying countries, and then we repeat our analysis with these selling and buying countries.

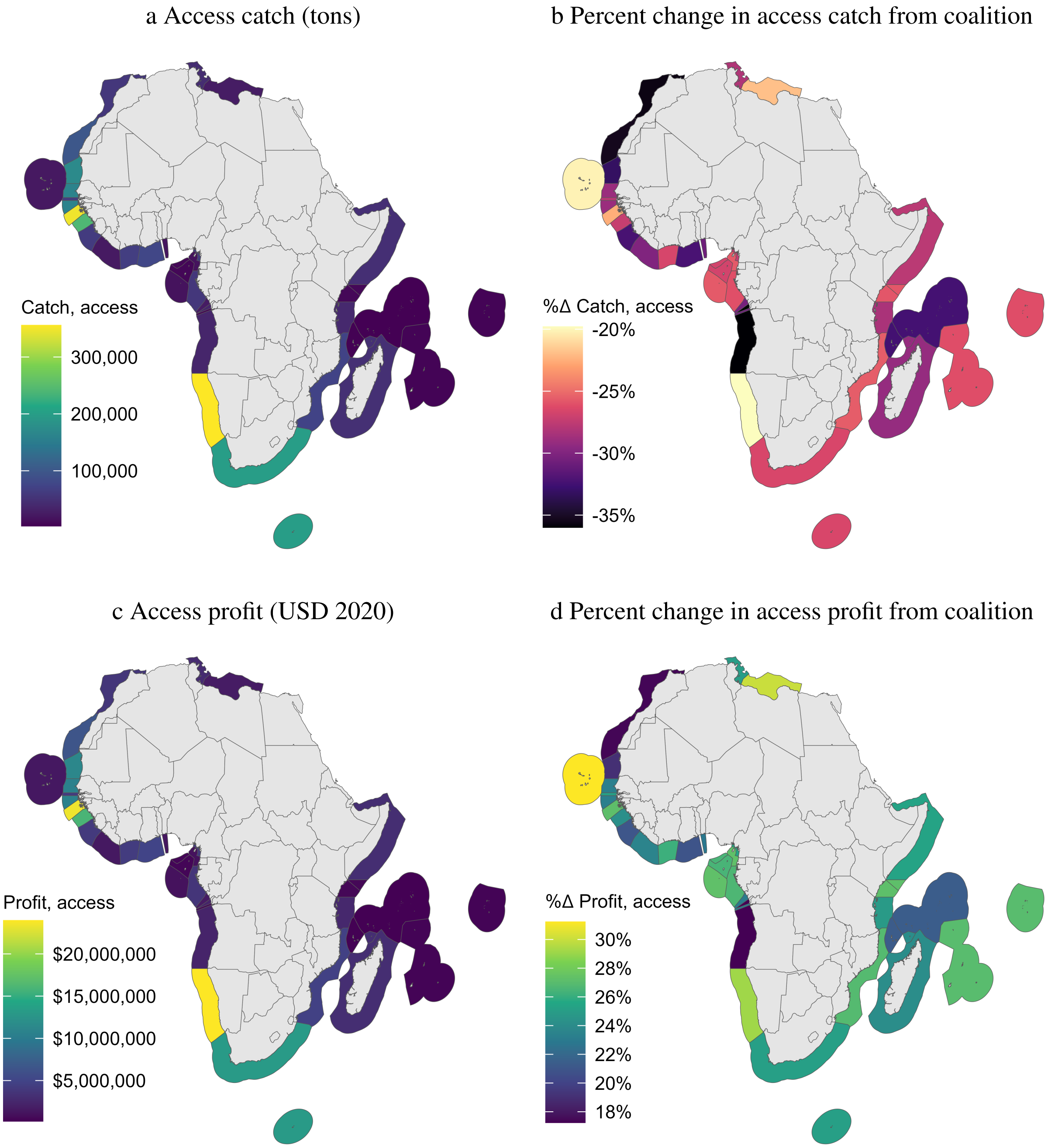

Figure S5: **Effect of Africa Coalition on access catch and profit by selling country when $\frac{b}{b_{MSY}} = 0.6$.** (a) Access catch and (c) profit are status quo values while (b) access catch and (d) profit display the percent changes under the coalition scenario relative to the status quo values. In our baseline specification we calculate each selling country's true status quo biomass with the assumption that $\frac{b}{b_{MSY}} = 0.8$ (Methods). As a robustness check, we set $\frac{b}{b_{MSY}} = 0.6$ and then we repeat our analysis.

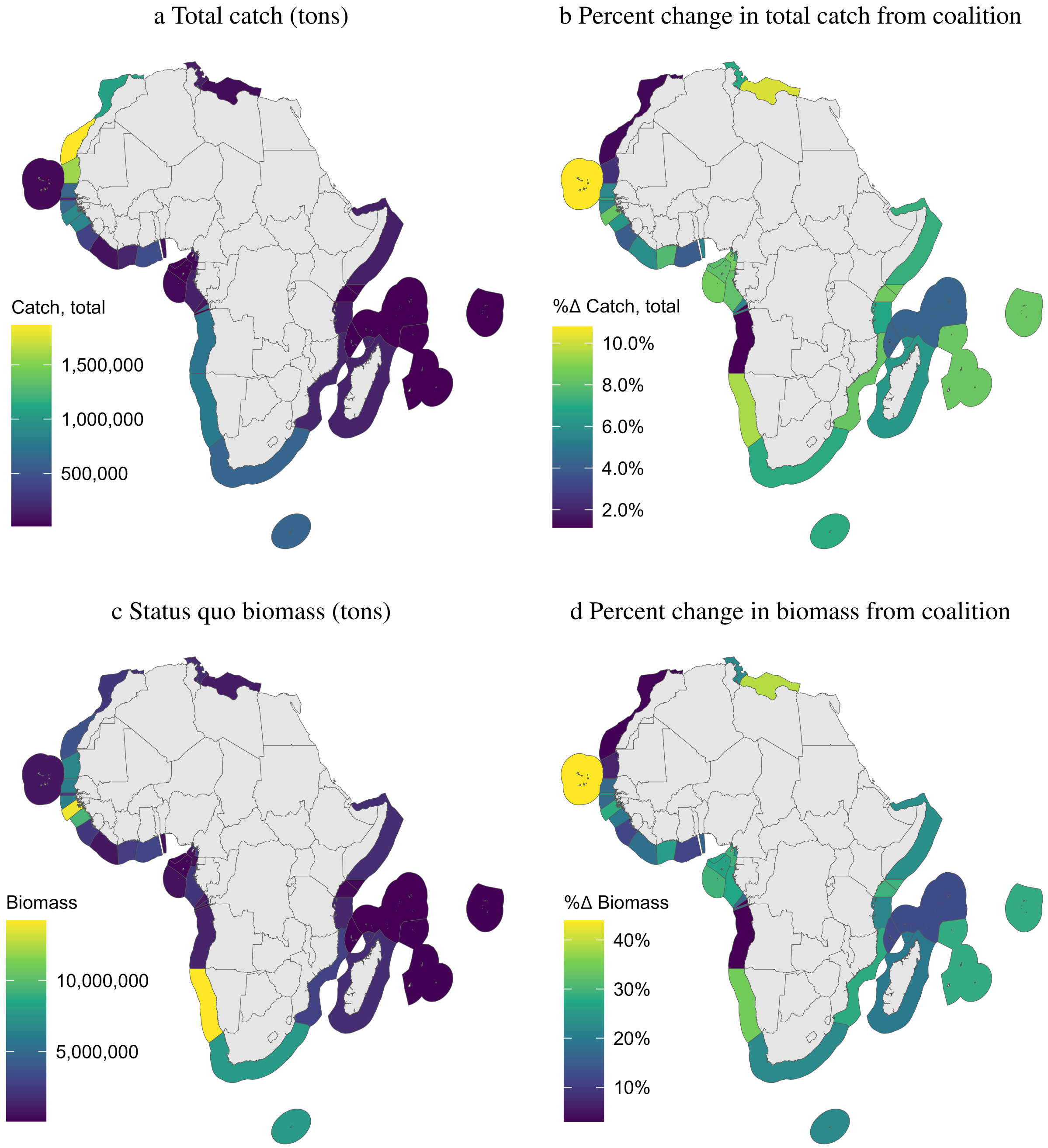

Figure S6: **Effect of Africa Coalition on total catch and biomass by selling country when $\frac{b}{b_{MSY}} = 0.6$.** (a) Total catch and (c) biomass are status quo values while (b) total catch and (d) biomass display the percent changes under the coalition scenario relative to the status quo values. In our baseline specification we calculate each selling country's true status quo biomass with the assumption that $\frac{b}{b_{MSY}} = 0.8$ (Methods). As a robustness check, we set $\frac{b}{b_{MSY}} = 0.6$ and then we repeat our analysis.

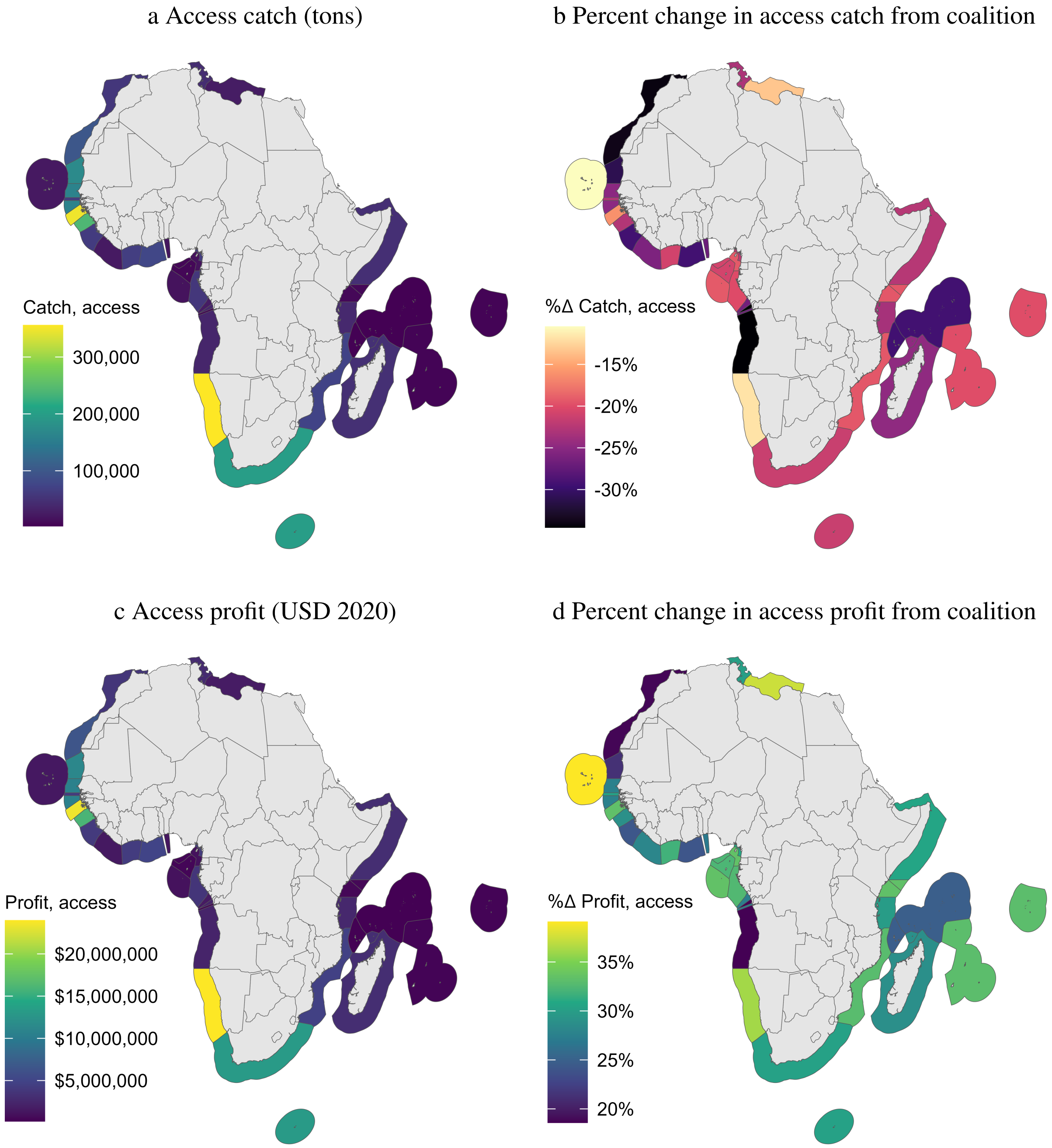

Figure S7: **Effect of Africa Coalition on access catch and profit by selling country when $\frac{b}{b_{MSY}} = 0.4$.** (a) Access catch and (c) profit are status quo values while (b) access catch and (d) profit display the percent changes under the coalition scenario relative to the status quo values. In our baseline specification we calculate each selling country's true status quo biomass with the assumption that $\frac{b}{b_{MSY}} = 0.8$ (Methods). As a robustness check, we set $\frac{b}{b_{MSY}} = 0.4$ and then we repeat our analysis.

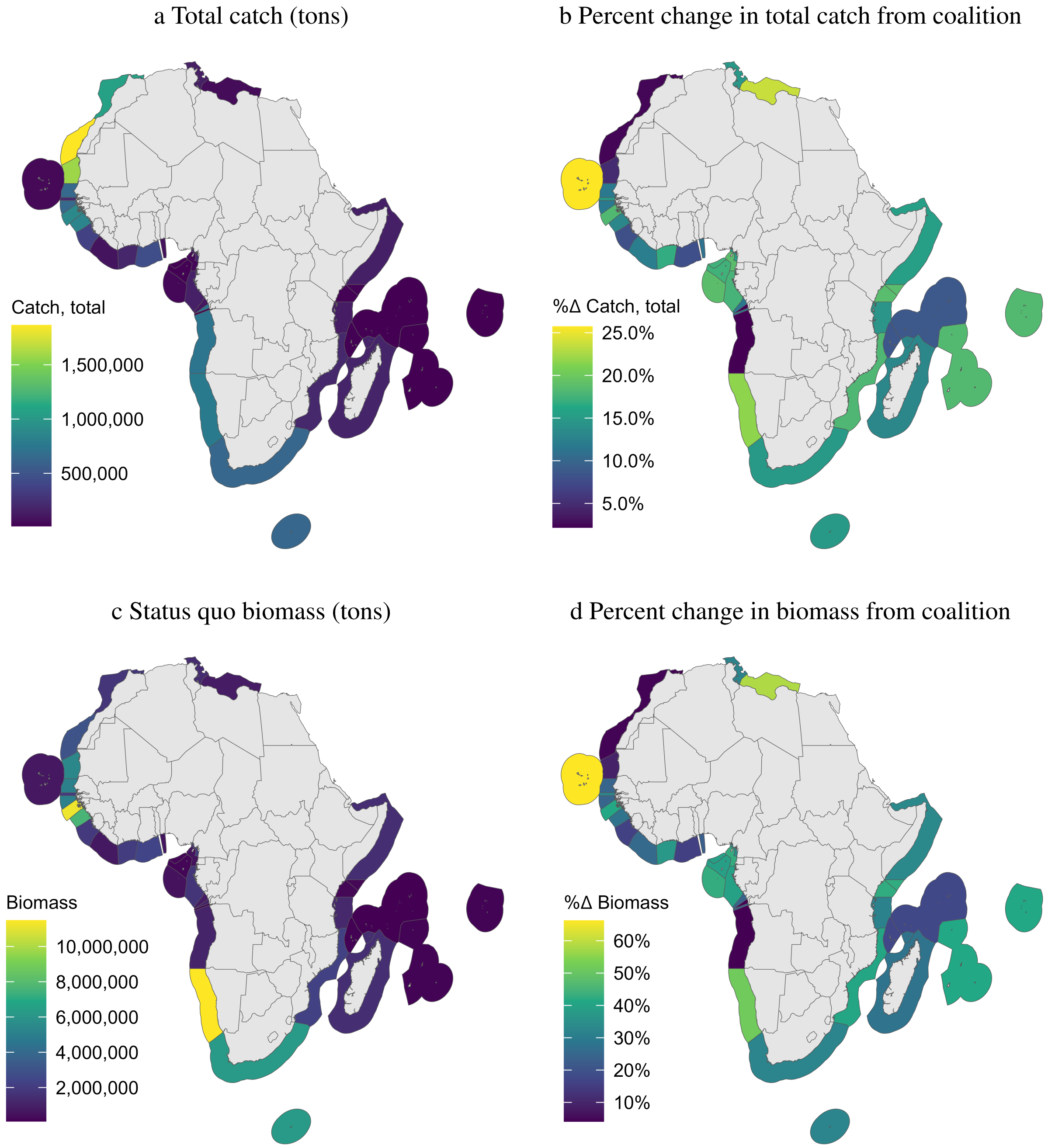

Figure S8: **Effect of Africa Coalition on total catch and biomass by selling country when** $\frac{b}{b_{MSY}} = 0.4$**.** (a) Total catch and (c) biomass are status quo values while (b) total catch and (d) biomass display the percent changes under the coalition scenario relative to the status quo values. In our baseline specification we calculate each selling country's true status quo biomass with the assumption that $\frac{b}{b_{MSY}} = 0.8$ (Methods). As a robustness check, we set $\frac{b}{b_{MSY}} = 0.4$ and then we repeat our analysis.

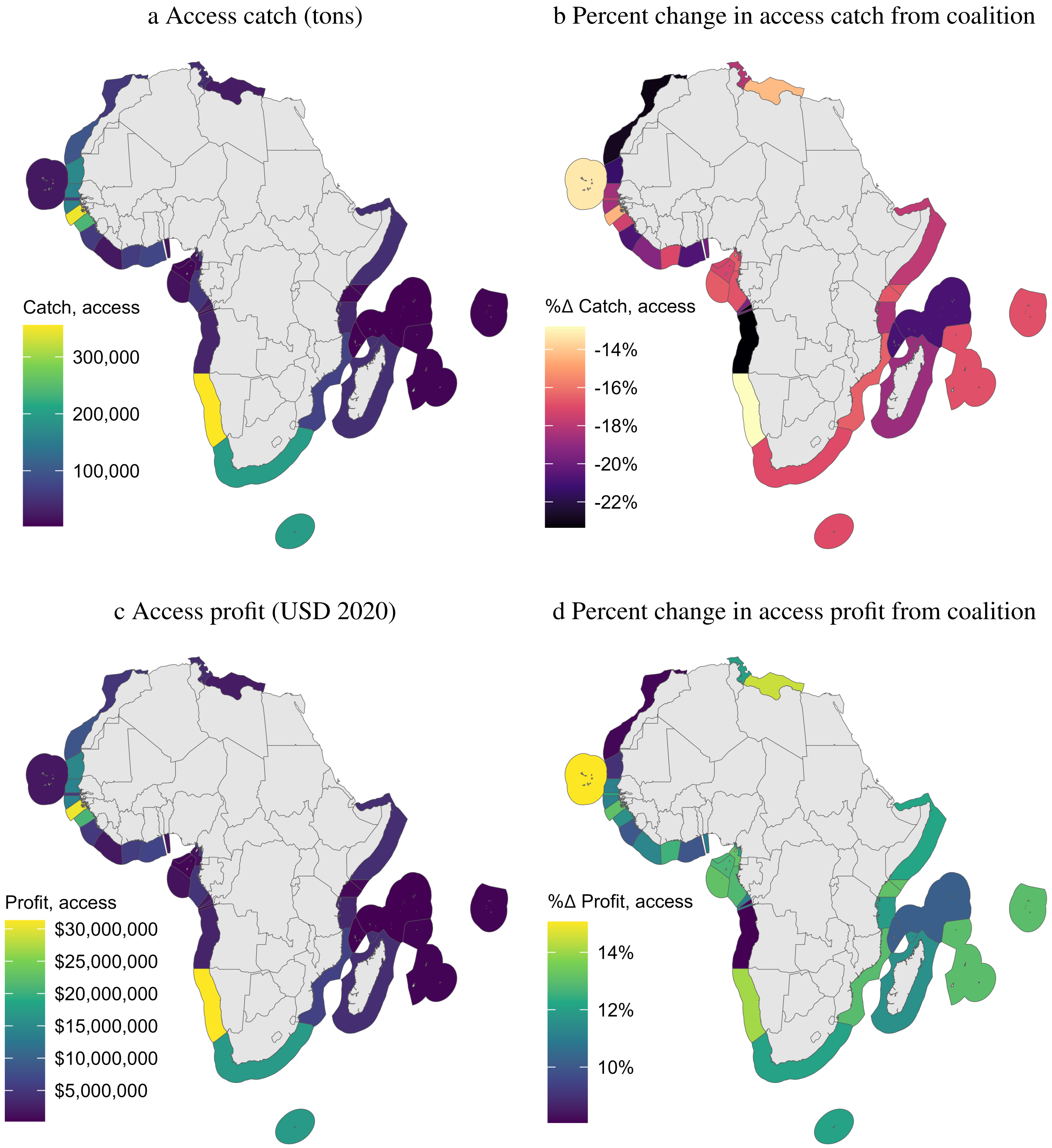

Figure S9: **Effect of Africa Coalition on access catch and profit by selling country when $\eta = 0.5$.** (a) Access catch and (c) profit are status quo values while (b) access catch and (d) profit display the percent changes under the coalition scenario relative to the status quo values. We assume $\eta = 1$ in our baseline specification (Methods). As a robustness check, we set $\eta = 0.5$ and then we repeat our analysis.

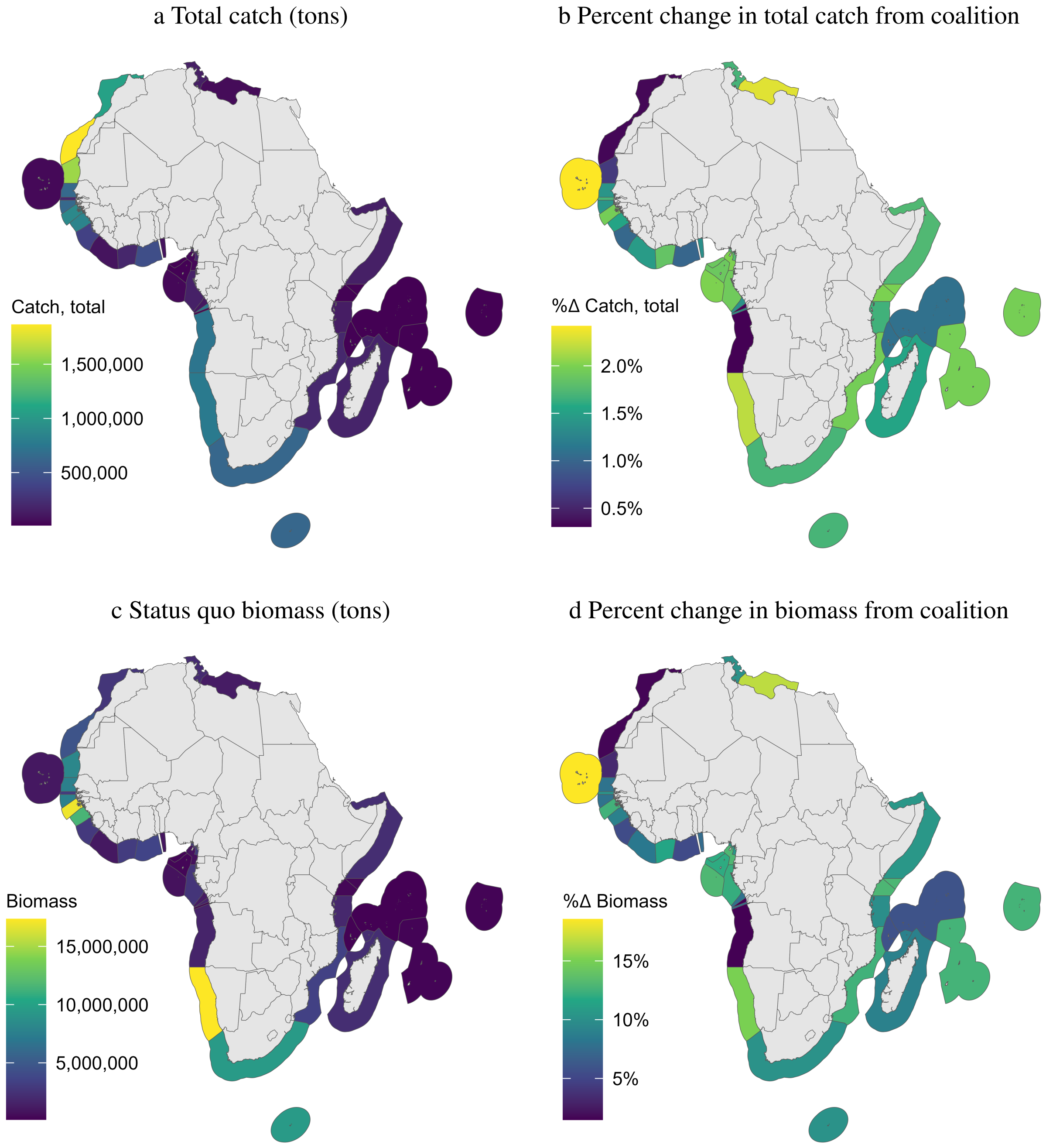

Figure S10: **Effect of Africa Coalition on total catch and biomass by selling country when $\eta = 0.5$.** (a) Total catch and (c) biomass are status quo values while (b) total catch and (d) biomass display the percent changes under the coalition scenario relative to the status quo values. We assume $\eta = 1$ in our baseline specification (Methods). As a robustness check, we set $\eta = 0.5$ and then we repeat our analysis.

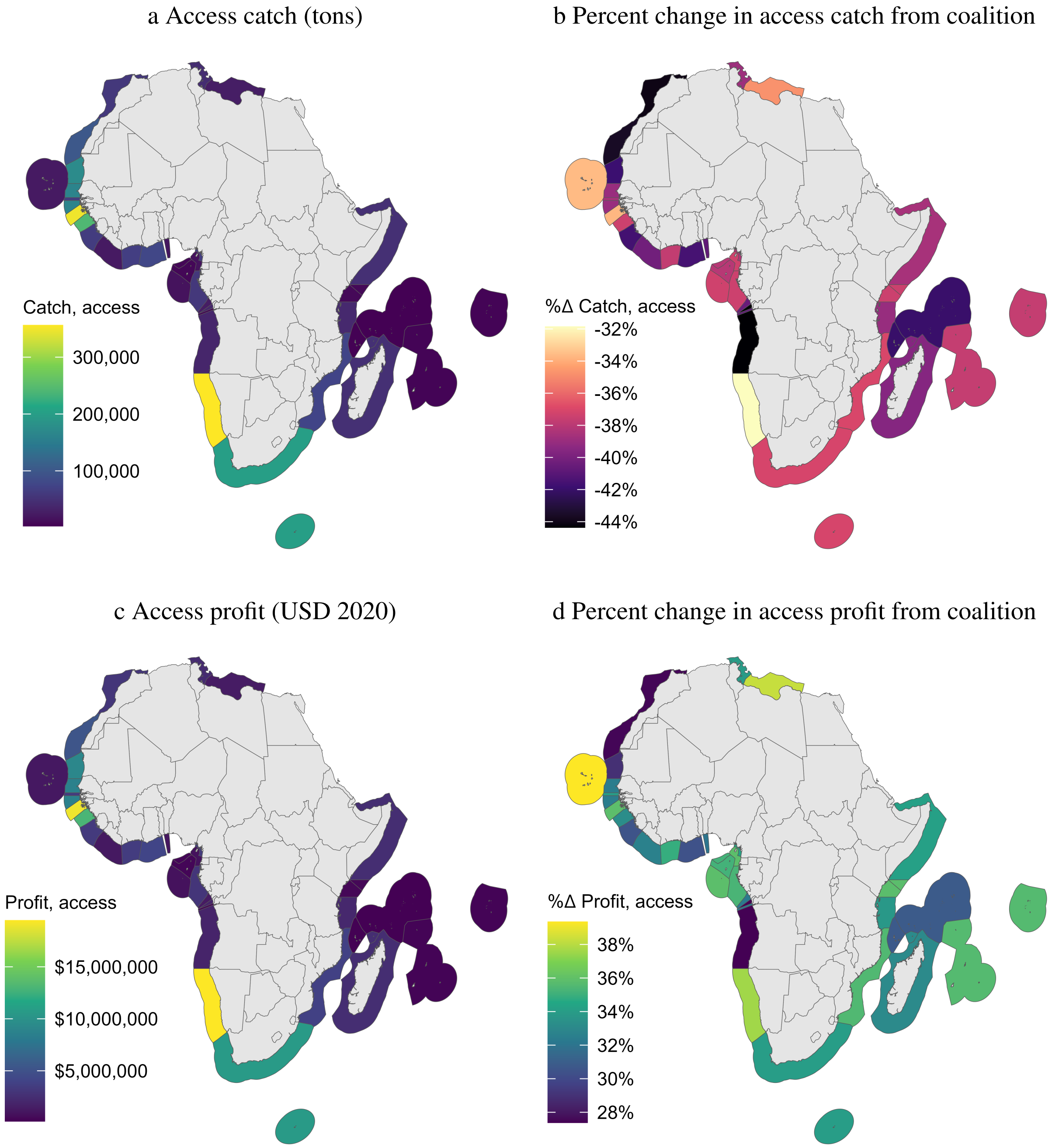

Figure S11: **Effect of Africa Coalition on access catch and profit by selling country when $\eta = 1.5$.** (a) Access catch and (c) profit are status quo values while (b) access catch and (d) profit display the percent changes under the coalition scenario relative to the status quo values. We assume $\eta = 1$ in our baseline specification (Methods). As a robustness check, we set $\eta = 1.5$ and then we repeat our analysis.

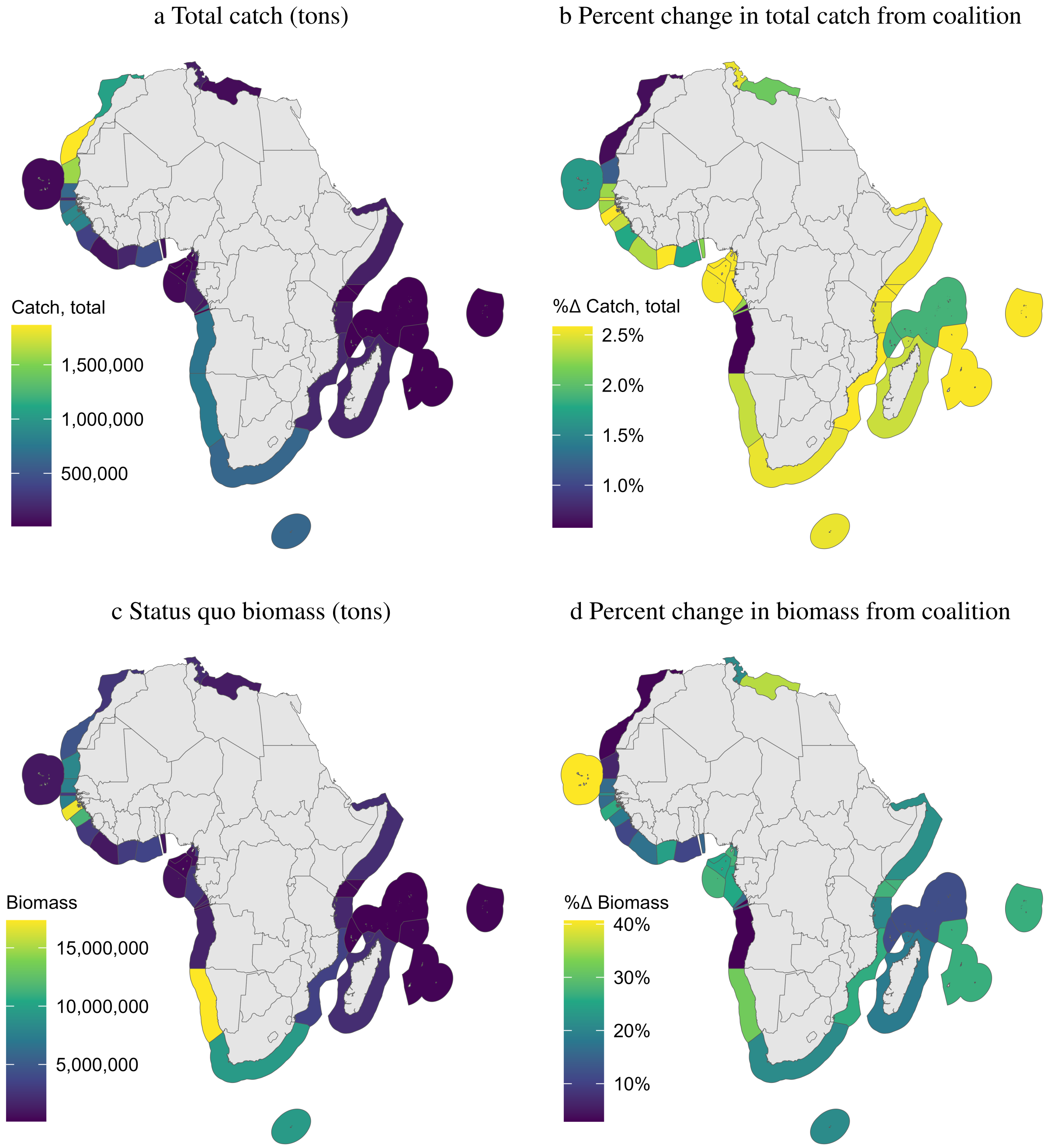

Figure S12: **Effect of Africa Coalition on total catch and biomass by selling country when $\eta = 1.5$.** (a) Total catch and (c) biomass are status quo values while (b) total catch and (d) biomass display the percent changes under the coalition scenario relative to the status quo values. We assume $\eta = 1$ in our baseline specification (Methods). As a robustness check, we set $\eta = 1.5$ and then we repeat our analysis.

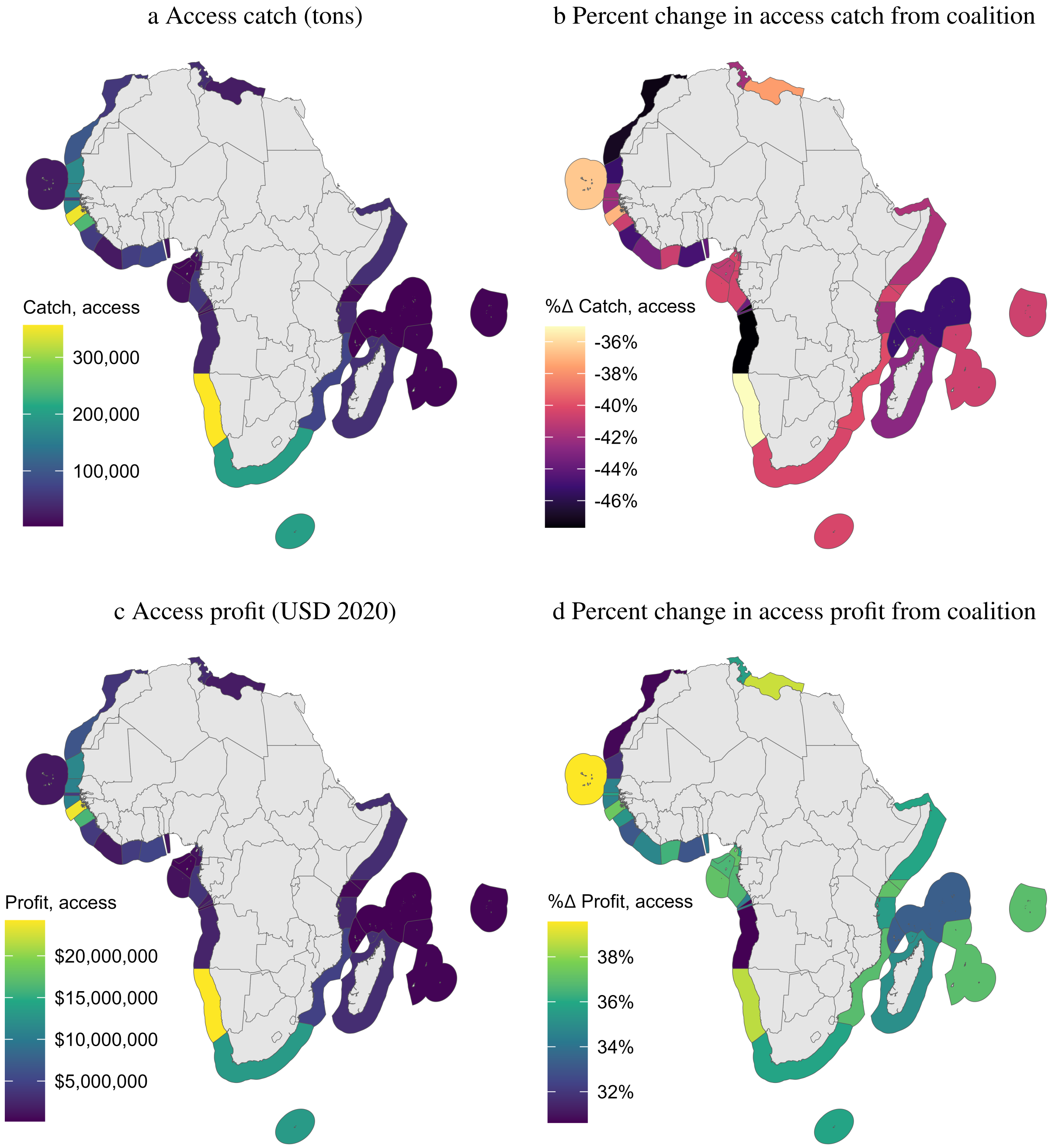

Figure S13: **Effect of Africa Coalition on access catch and profit by selling country when $\epsilon = 1.5$.** (a) Access catch and (c) profit are status quo values while (b) access catch and (d) profit display the percent changes under the coalition scenario relative to the status quo values. We assume $\epsilon = 2$ in our baseline specification (Methods). As a robustness check, we set $\epsilon = 1.5$ and then we repeat our analysis.

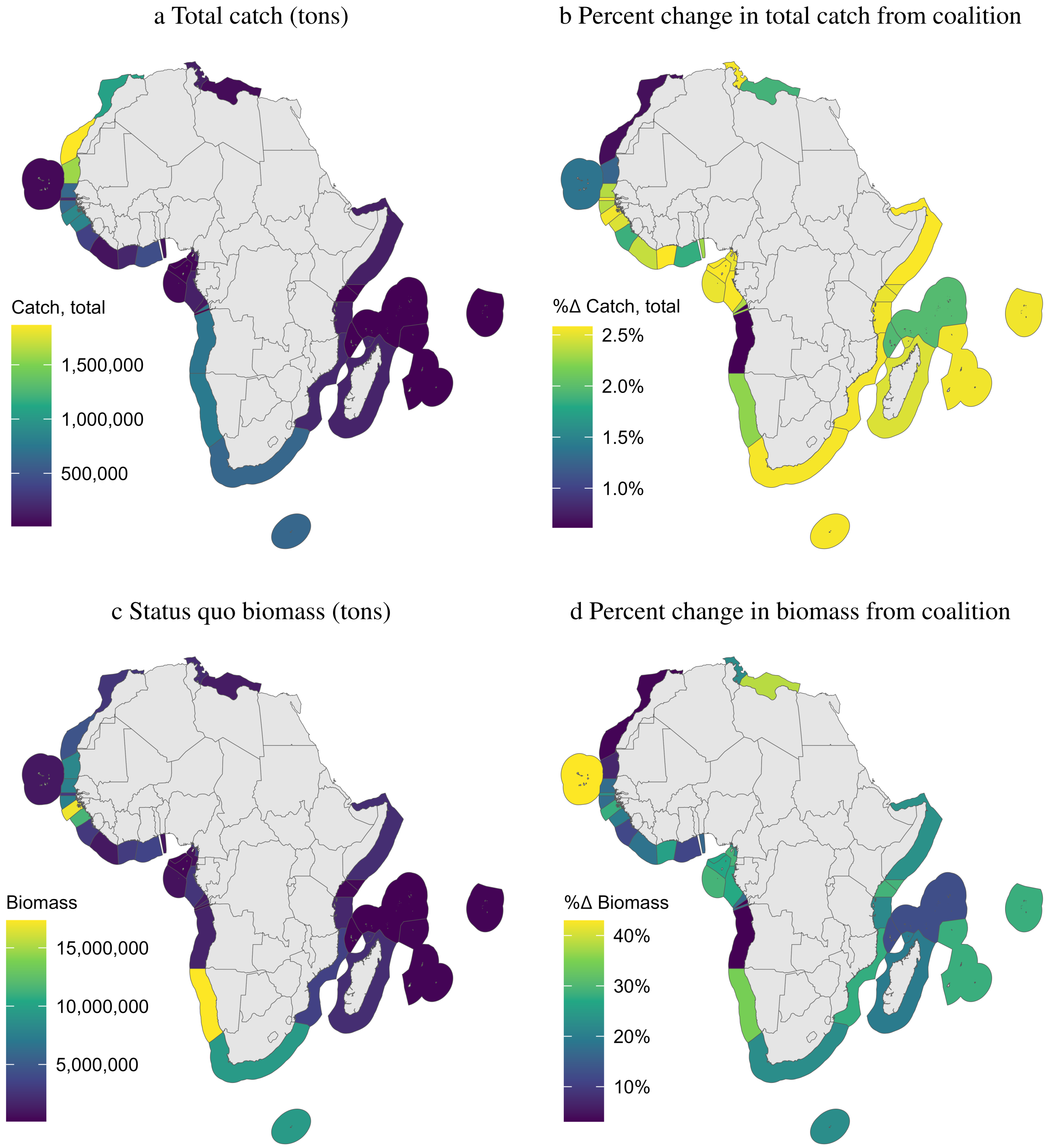

Figure S14: **Effect of Africa Coalition on total catch and biomass by selling country when $\epsilon = 1.5$.** (a) Total catch and (c) biomass are status quo values while (b) total catch and (d) biomass display the percent changes under the coalition scenario relative to the status quo values. We assume $\epsilon = 2$ in our baseline specification (Methods). As a robustness check, we set $\epsilon = 1.5$ and then we repeat our analysis.

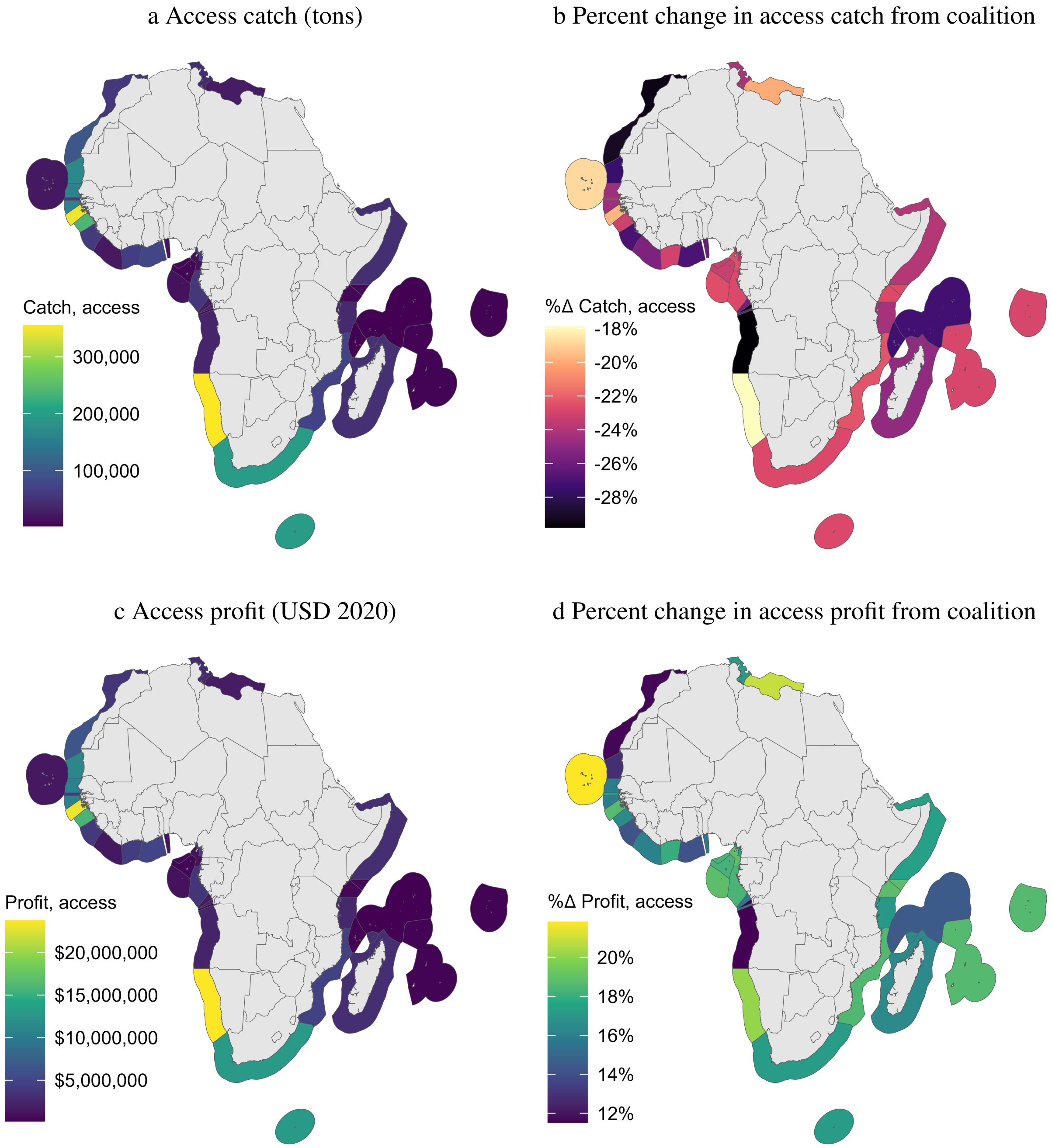

Figure S15: **Effect of Africa Coalition on access catch and profit by selling country when $\epsilon = 2.5$.** (a) Access catch and (c) profit are status quo values while (b) access catch and (d) profit display the percent changes under the coalition scenario relative to the status quo values. We assume $\epsilon = 2$ in our baseline specification (Methods). As a robustness check, we set $\epsilon = 2.5$ and then we repeat our analysis.

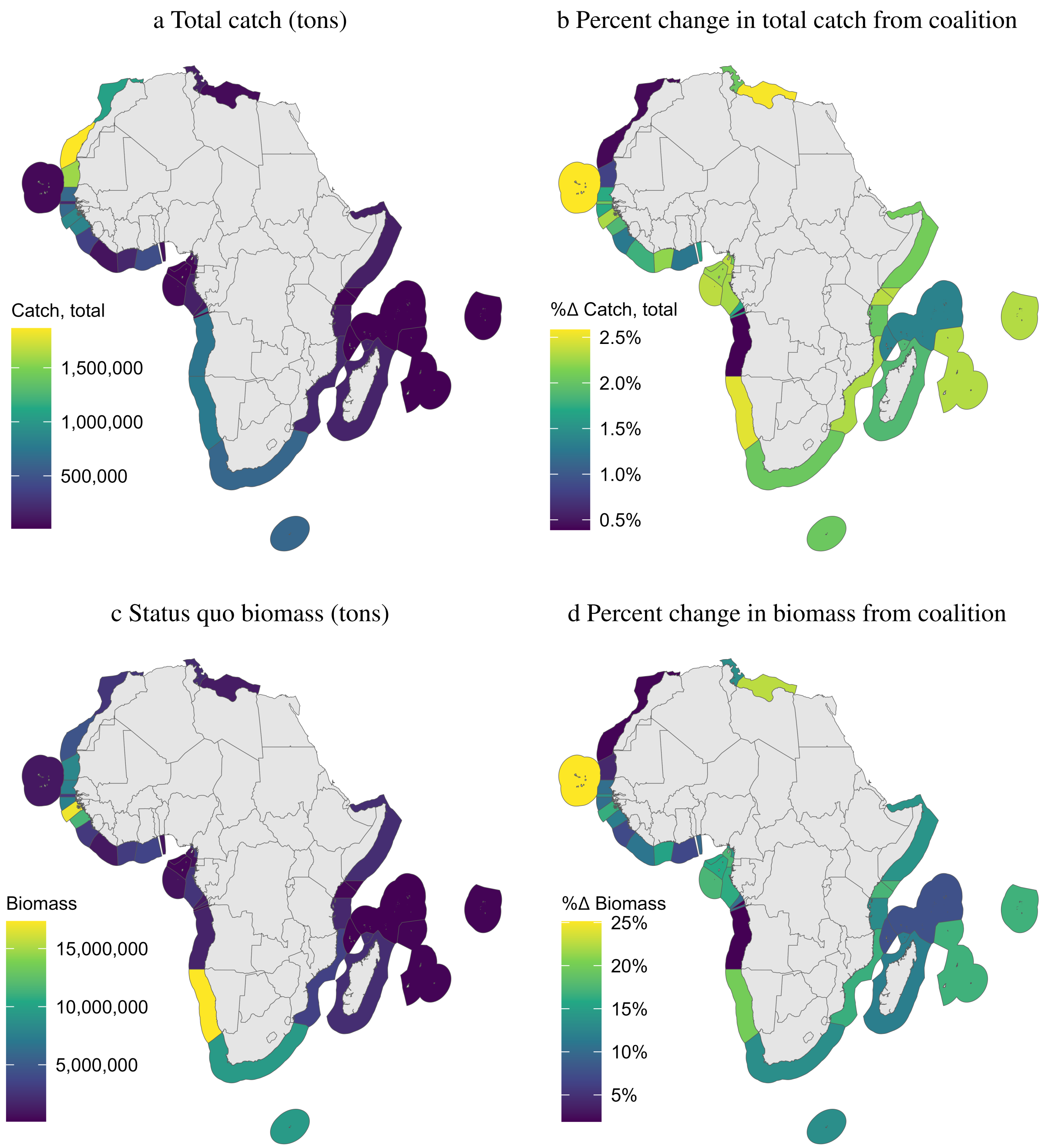

Figure S16: **Effect of Africa Coalition on total catch and biomass by selling country when $\epsilon = 2.5$.** (a) Total catch and (c) biomass are status quo values while (b) total catch and (d) biomass display the percent changes under the coalition scenario relative to the status quo values. We assume $\epsilon = 2$ in our baseline specification (Methods). As a robustness check, we set $\epsilon = 2.5$ and then we repeat our analysis.

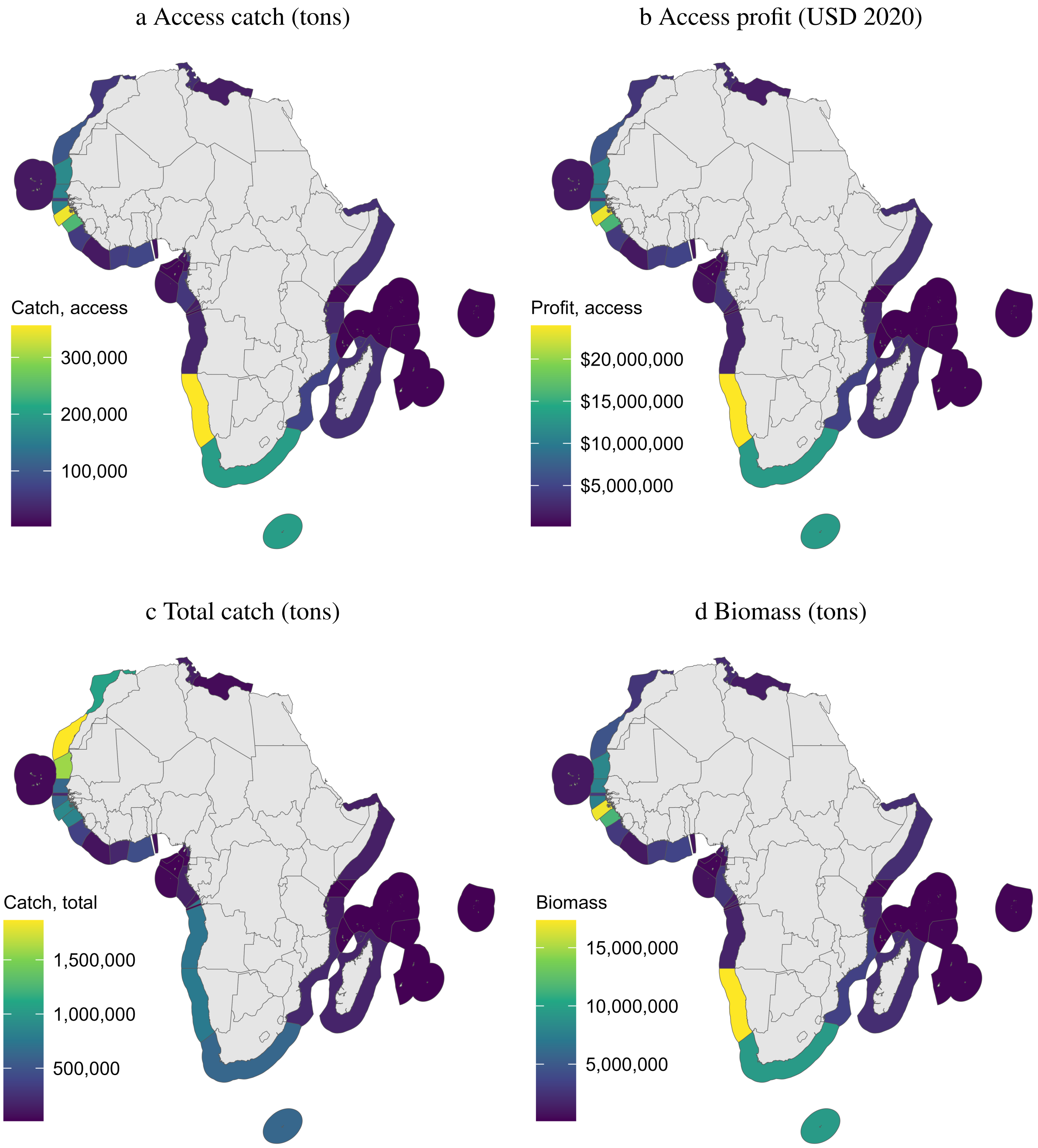

Figure S17: **Status quo (a) access catch, (b) profit, (c) total catch, and (d) biomass values by selling country.**

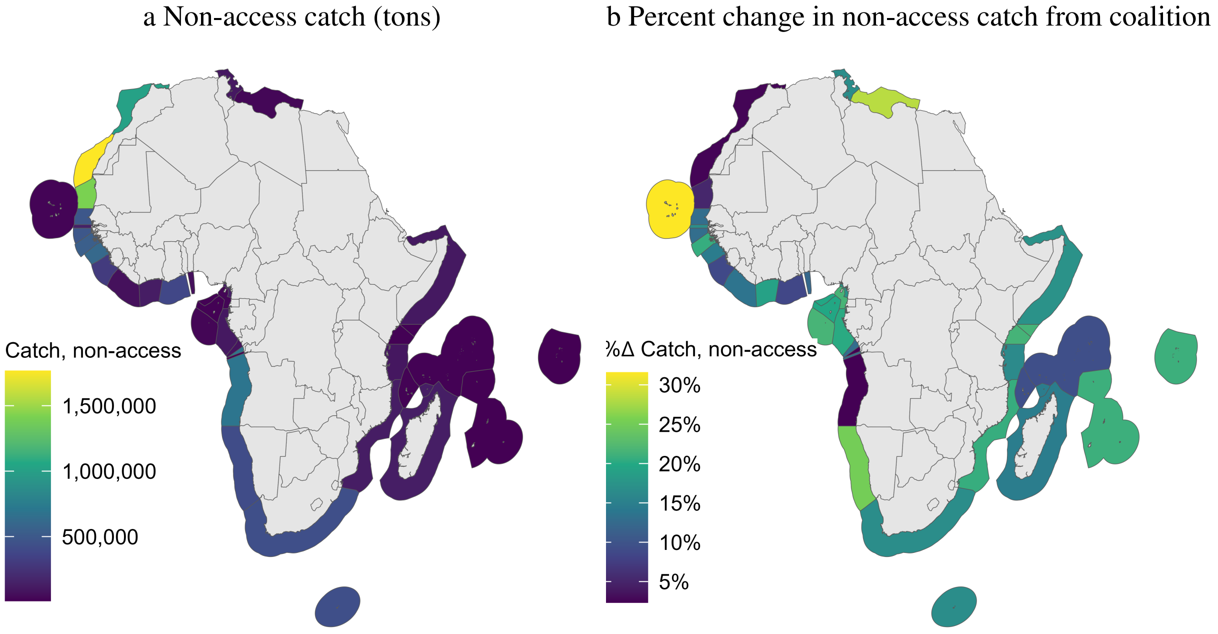

Figure S18: **Effect of Africa Coalition on non-access catch.** (a) Status quo values and (b) percent change in non-access catch under Africa Coalition relative to status quo values.

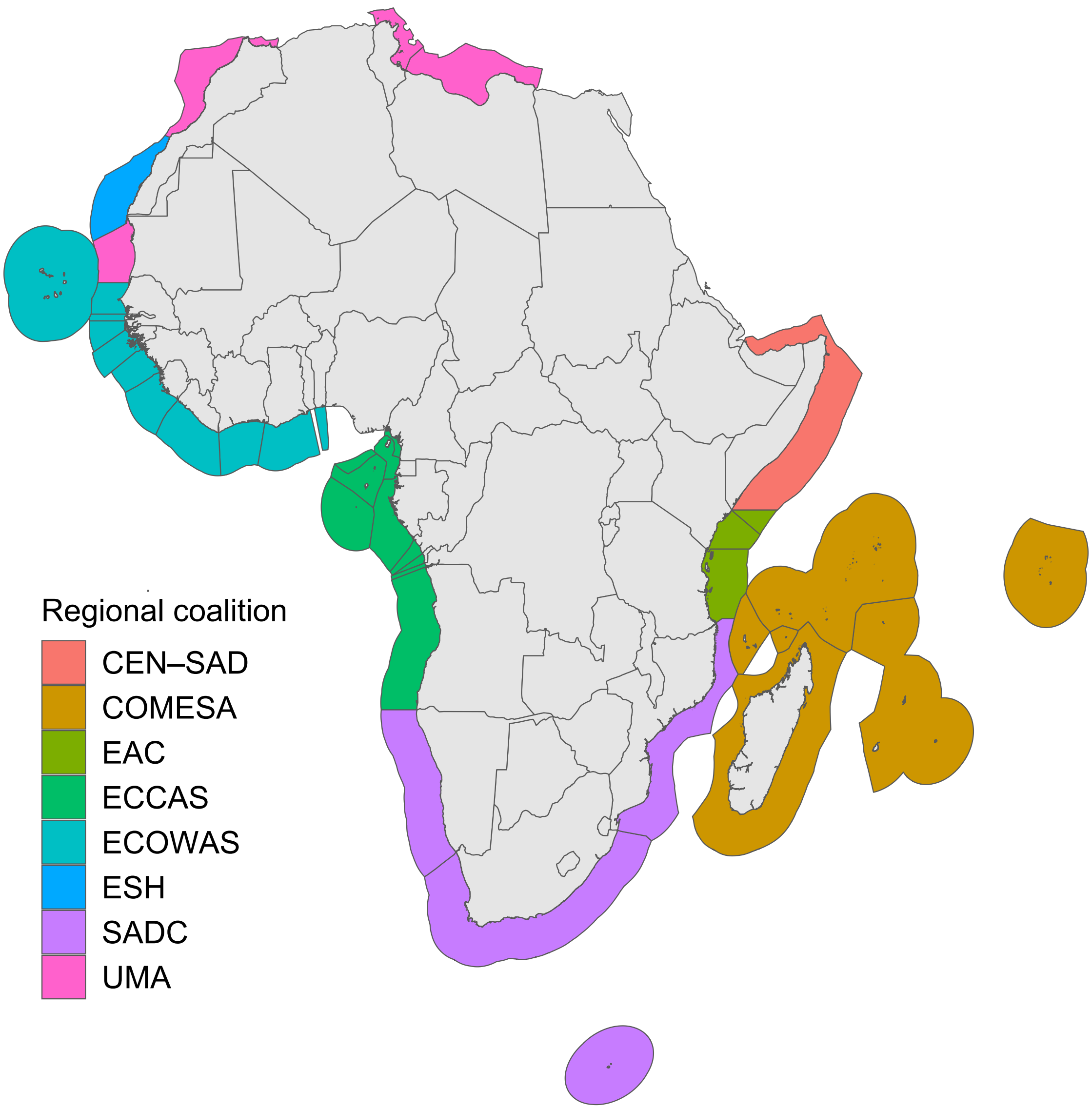

Figure S19: **Grouping of selling countries into regional coalitions for the regional coalition scenario.** We apply an iterative decision rule to map each selling country to one of the Regional Economic Communities (RECs) recognized by the African Union: the Community of Sahel-Saharan States (CEN-SAD), the Common Market for Eastern and Southern Africa (COMESA), the East African Community (EAC), the Economic Community of Central African States (ECCAS), the Economic Community of West African States (ECOWAS), the Southern African Development Community (SADC), and the Arab Maghreb Union (UMA). Western Sahara (ESH) does not belong to any REC so we do not include it in any regional coalition.

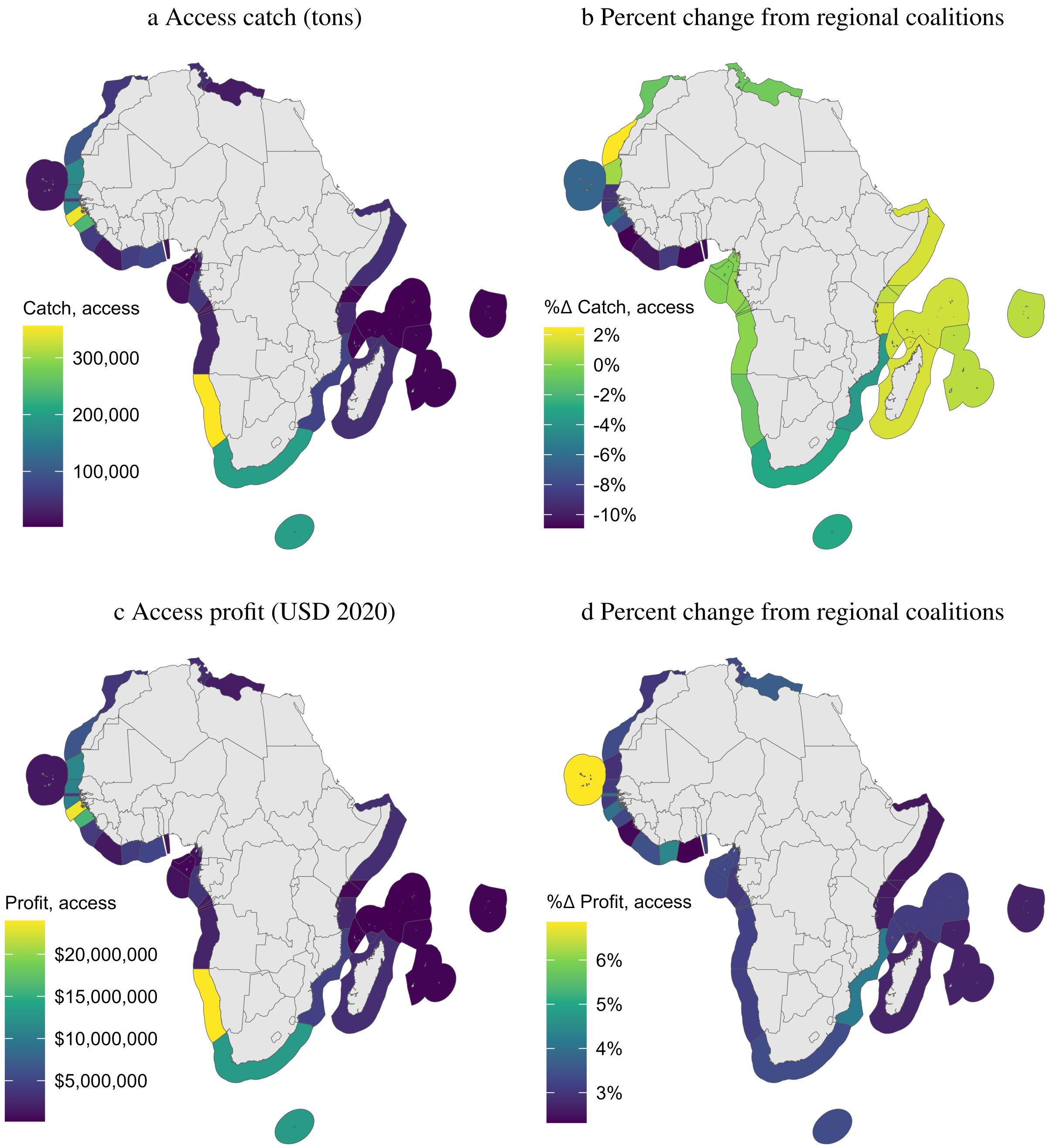

Figure S20: **Effect of regional coalitions on access catch and profit by selling country.** (a) Access catch and (c) profit are status quo values while (b) access catch and (d) profit display the percent changes under the regional coalitions scenario relative to the status quo values.

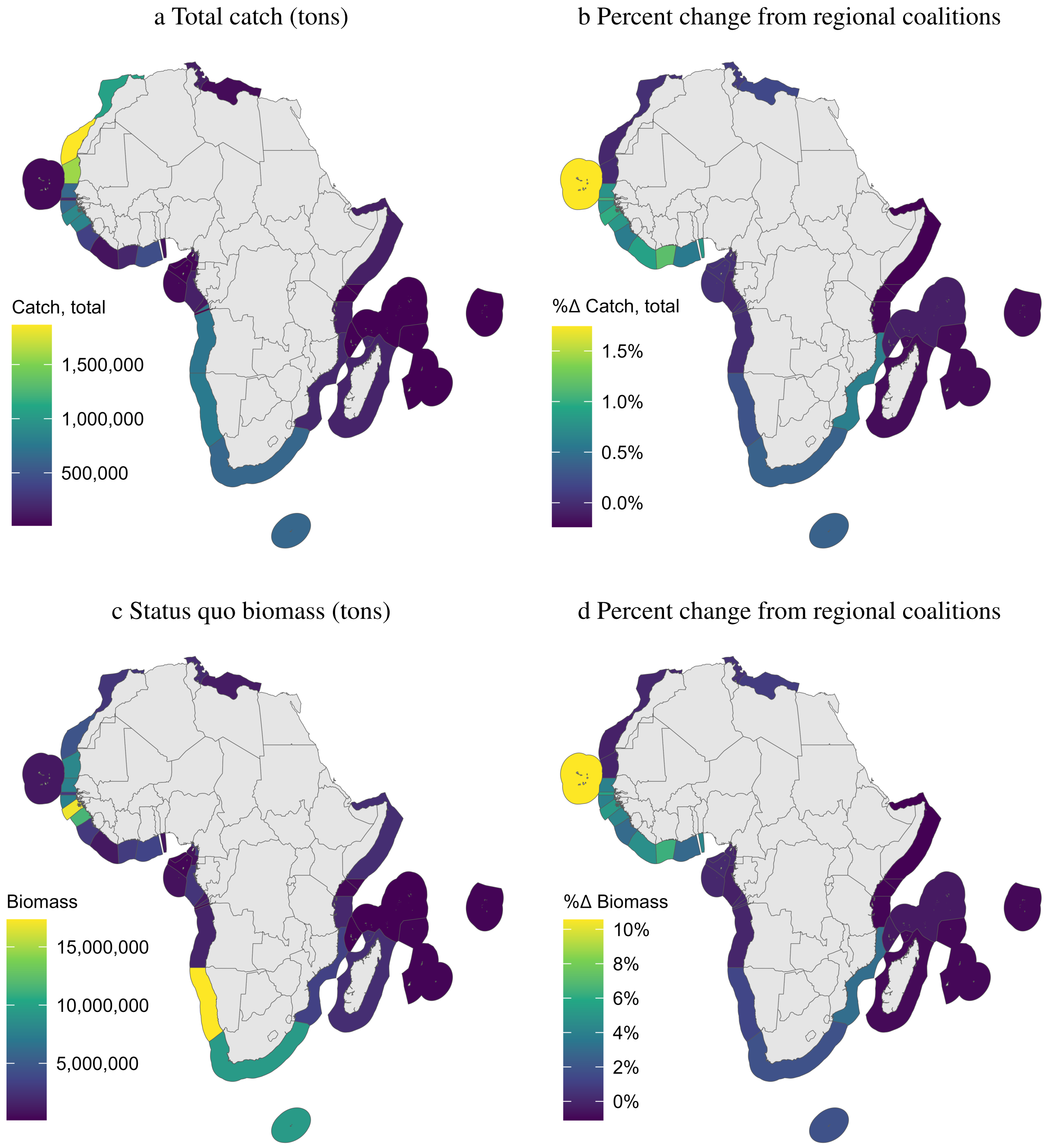

Figure S21: **Effect of regional coalitions on total catch and biomass by selling country.** (a) Total catch and (c) biomass are status quo values while (b) total catch and (d) biomass display the percent changes under the regional coalitions scenario relative to the status quo values.

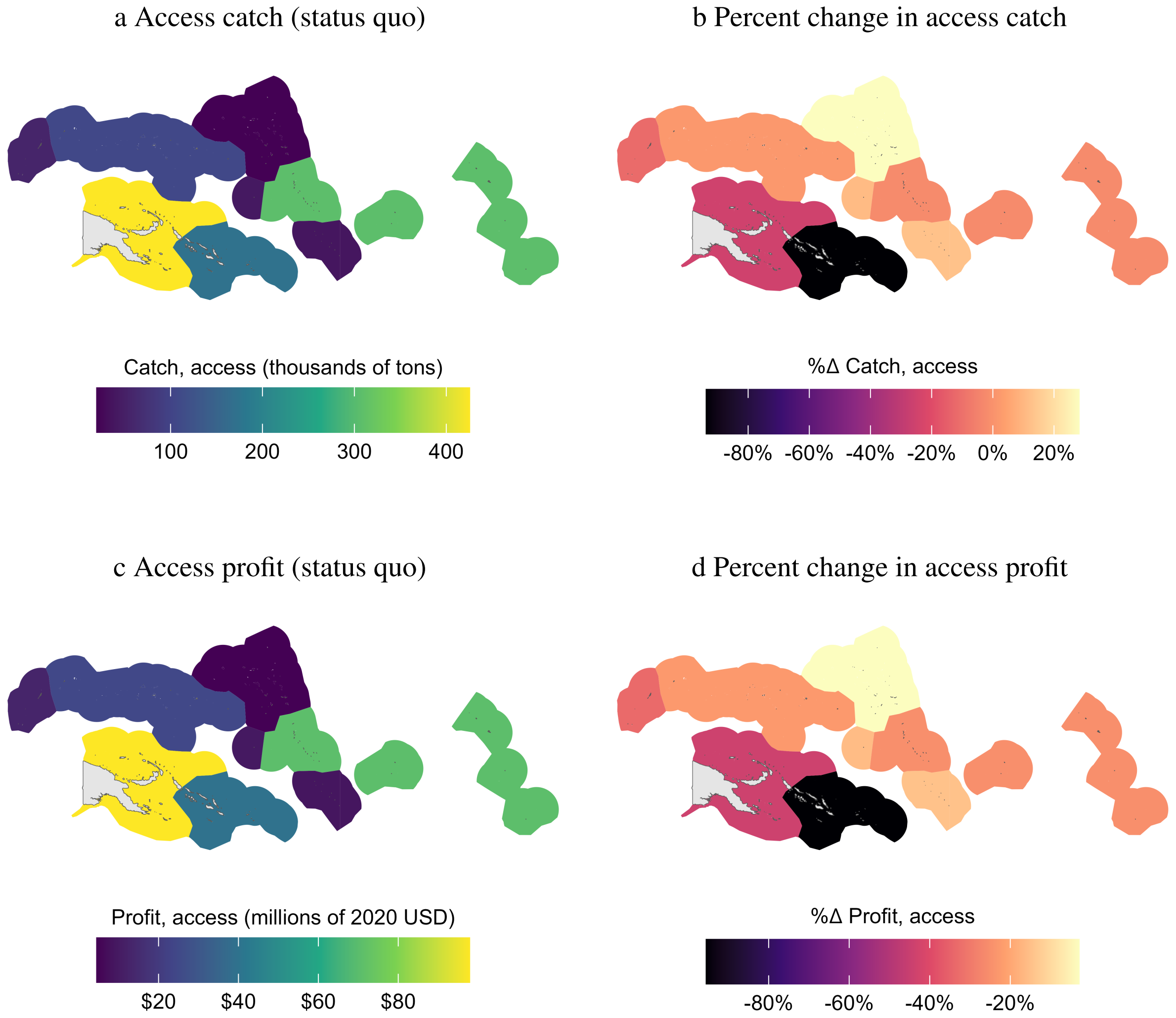

Figure S22: **Access catch and profit if PNA coalition did not exist.** (a) Access catch and (c) access profit by selling country in the status quo scenario that the Parties to the Nauru Agreement (PNA) coalition exists; percent change in (b) access catch and (d) access profit if the PNA coalition did not exist. Percent change is relative to status quo value. The large effects for the Solomon Islands occur because its status quo total catch policy function is steep (Methods).

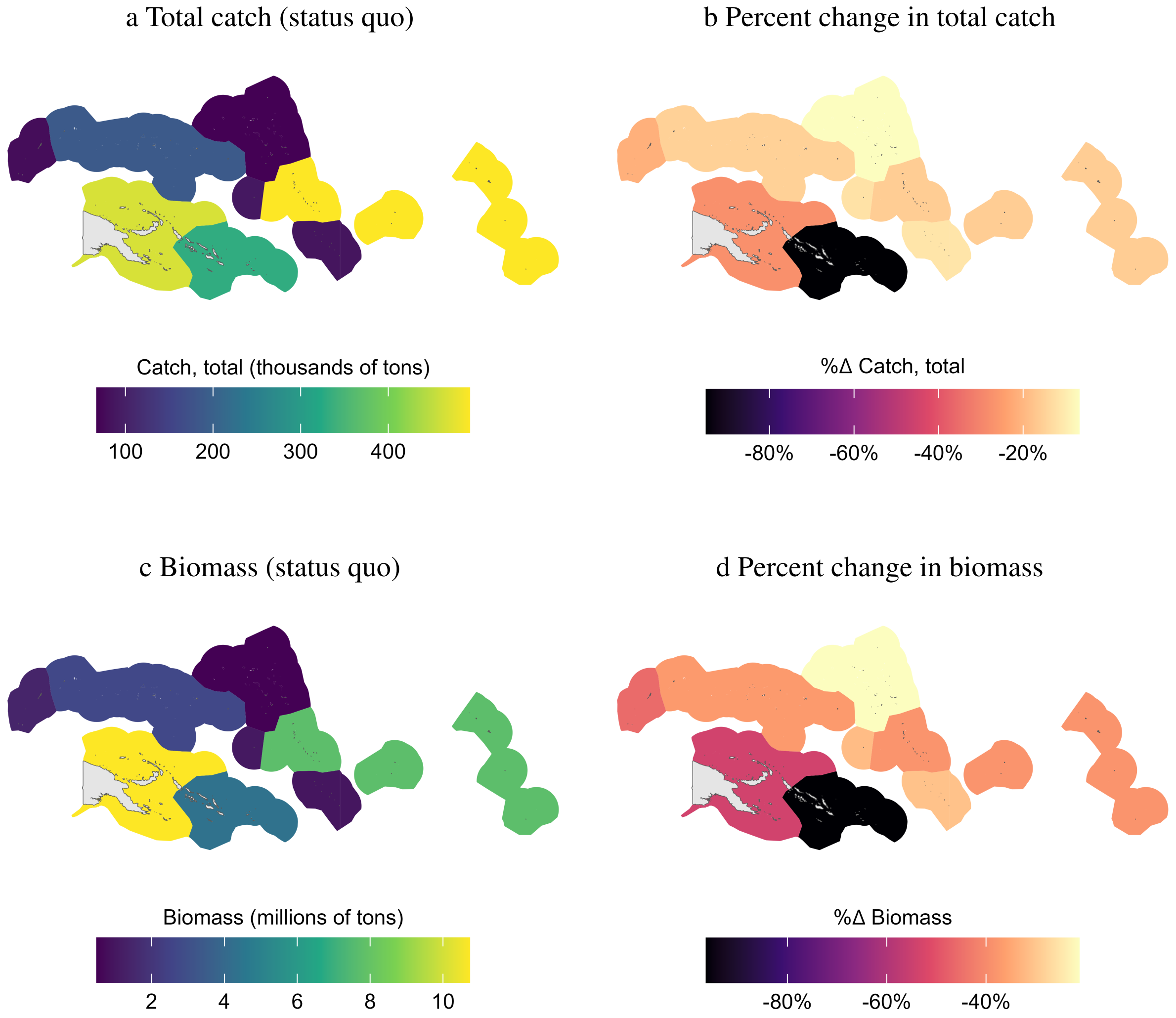

Figure S23: **Total catch and biomass if PNA did not exist.** (a) Total catch (access plus non-access catch) and (c) biomass by selling country in the status quo scenario that the Parties to the Nauru Agreement (PNA) coalition exists; percent change in (b) total catch and (d) biomass if the PNA coalition did not exist. Percent change is relative to status quo value. The large effects for the Solomon Islands occur because its status quo total catch policy function is steep (Methods).

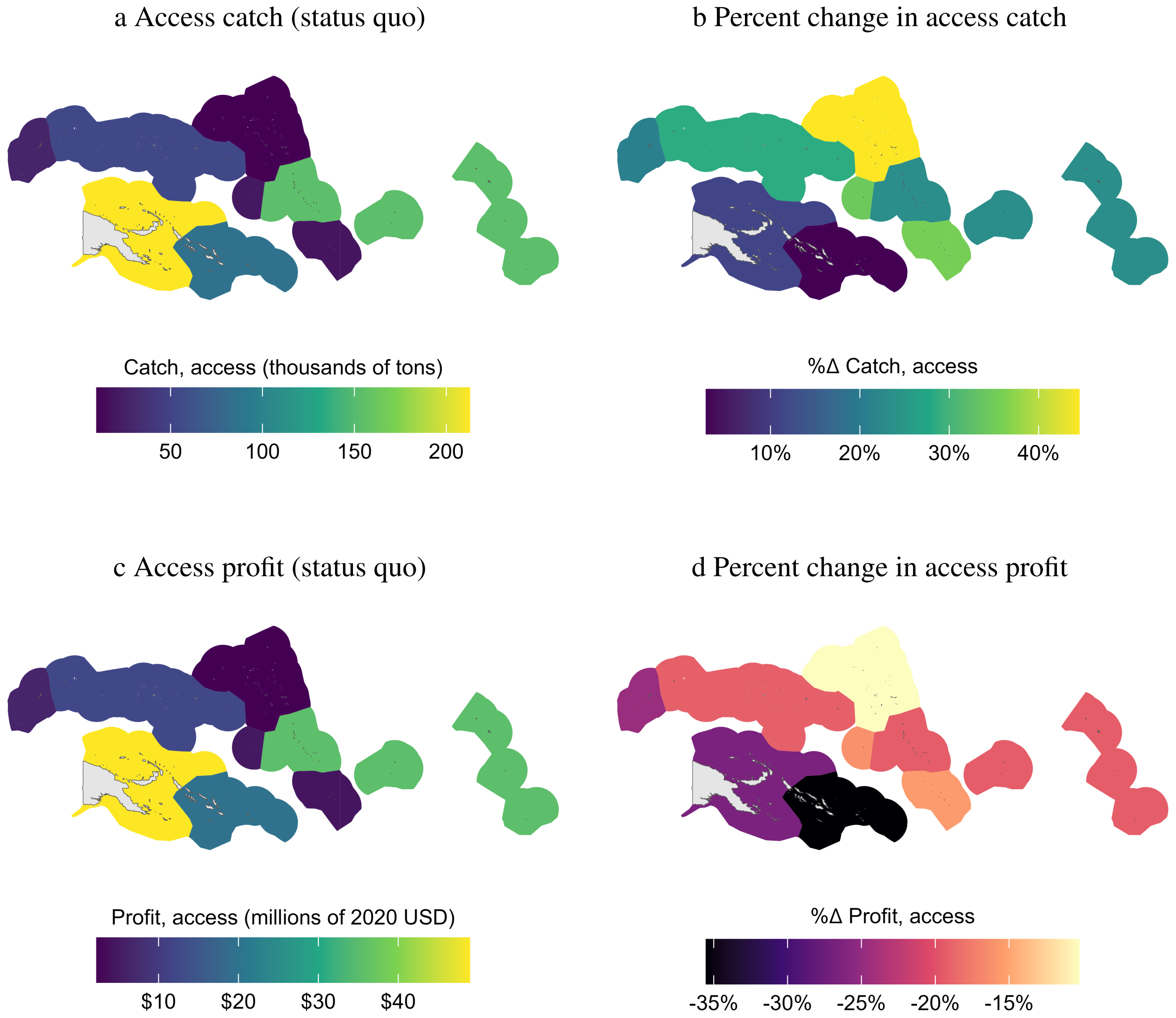

Figure S24: **Access catch and profit if PNA coalition did not exist when access catch is half of foreign catch.** (a) Access catch and (c) access profit by selling country in the status quo scenario that the Parties to the Nauru Agreement (PNA) coalition exists; percent change in (b) access catch and (d) access profit if the PNA coalition did not exist. Percent change is relative to status quo value. Instead of assuming access catch equals foreign catch, in this robustness check we assume access catch is half of foreign catch and then we repeat our analysis.

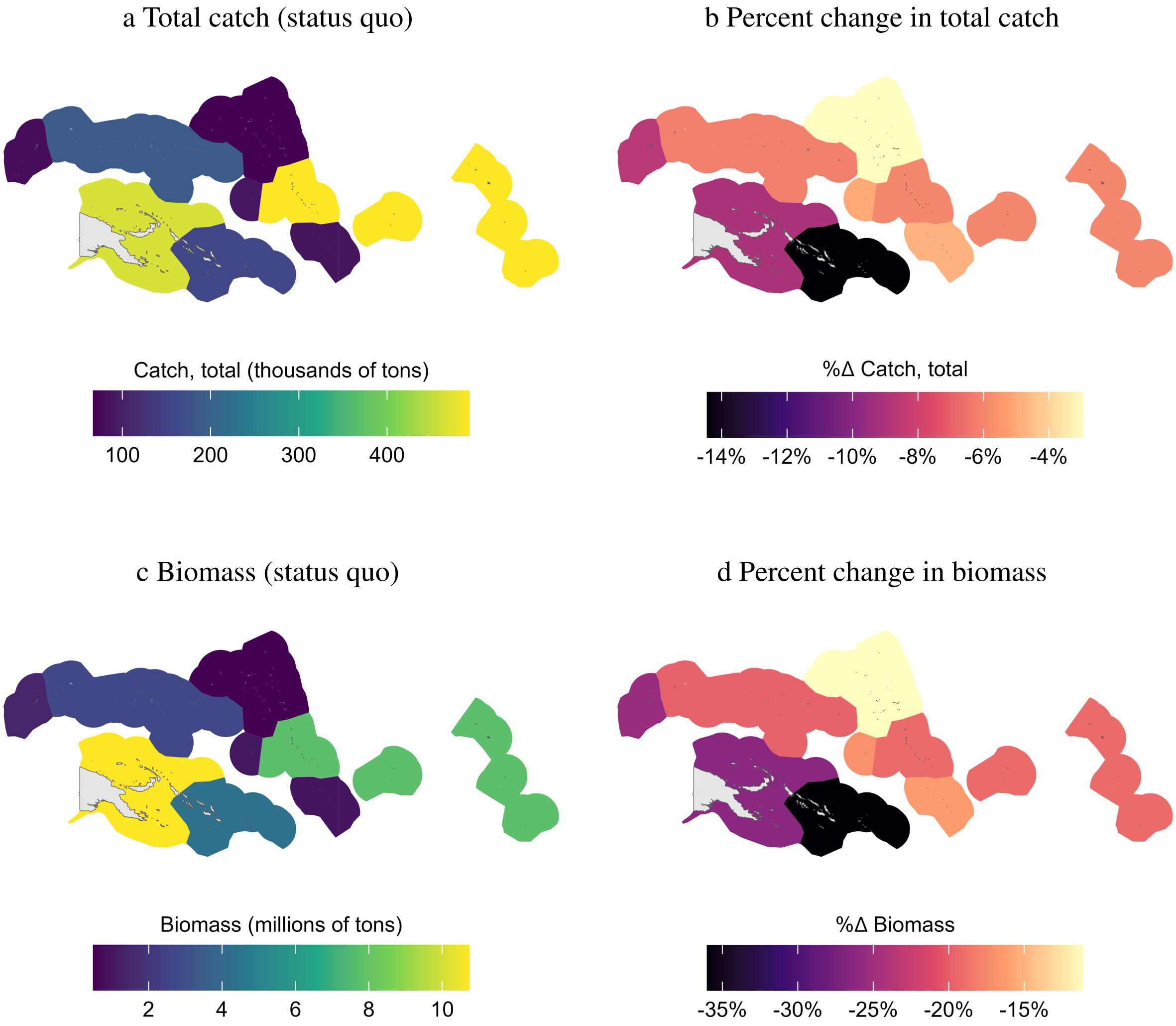

Figure S25: **Total catch and biomass if PNA did not exist when access catch is half of foreign catch.** (a) Total catch (access plus non-access catch) and (c) biomass by selling country in the status quo scenario that the Parties to the Nauru Agreement (PNA) coalition exists; percent change in (b) total catch and (d) biomass if the PNA coalition did not exist. Percent change is relative to status quo value. Instead of assuming access catch equals foreign catch, in this robustness check we assume access catch is half of foreign catch and then we repeat our analysis.

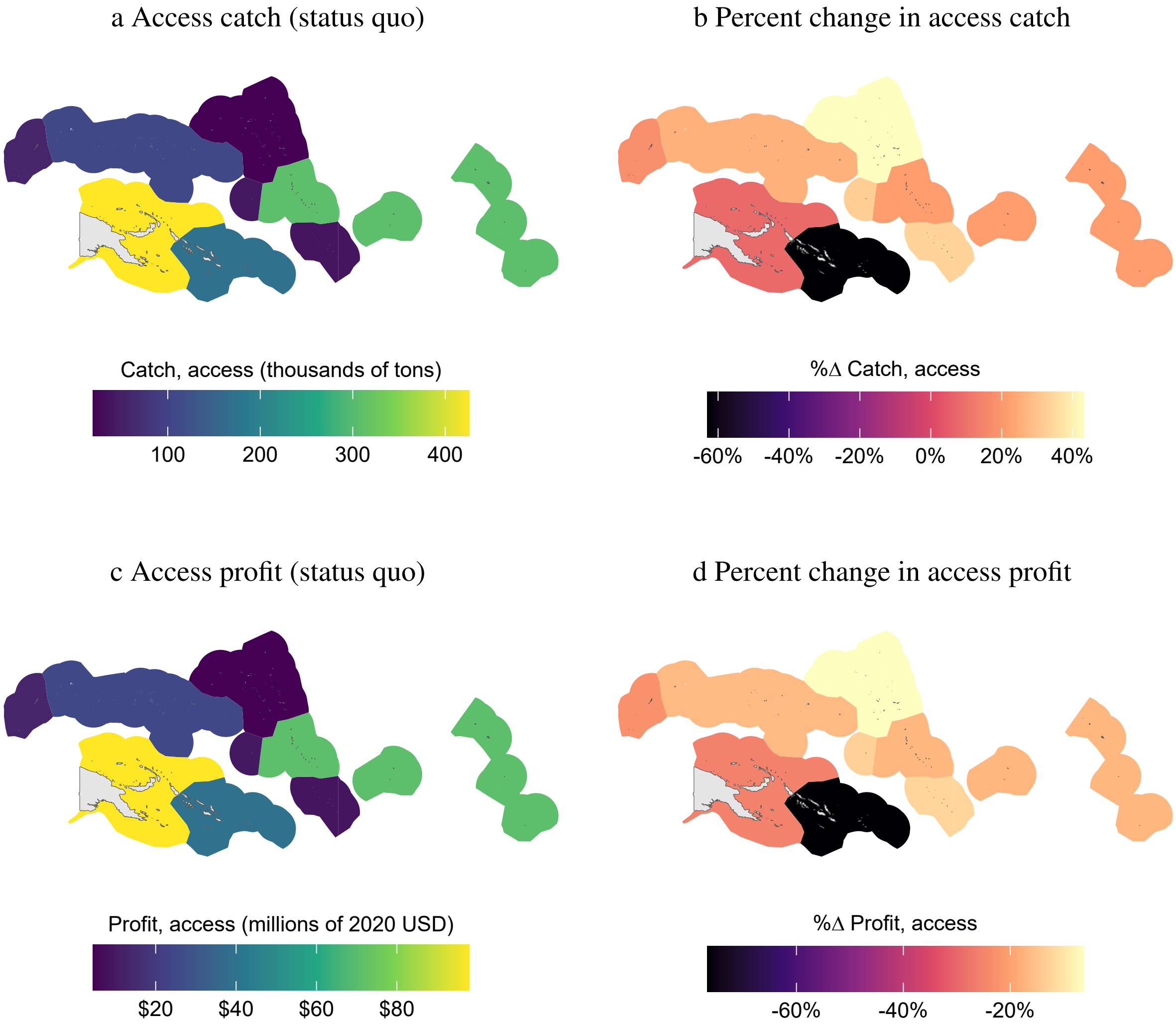

Figure S26: **Access catch and profit if PNA coalition did not exist when** $\frac{b}{b_{MSY}} = 1.3$**.** (a) Access catch and (c) access profit by selling country in the status quo scenario that the Parties to the Nauru Agreement (PNA) coalition exists; percent change in (b) access catch and (d) access profit if the PNA coalition did not exist. Percent change is relative to status quo value. In our baseline specification we calculate each selling country's true status quo biomass with the assumption that $\frac{b}{b_{MSY}} = 0.8$ (Methods). As a robustness check, we set $\frac{b}{b_{MSY}} = 1.3$ and then we repeat our analysis.

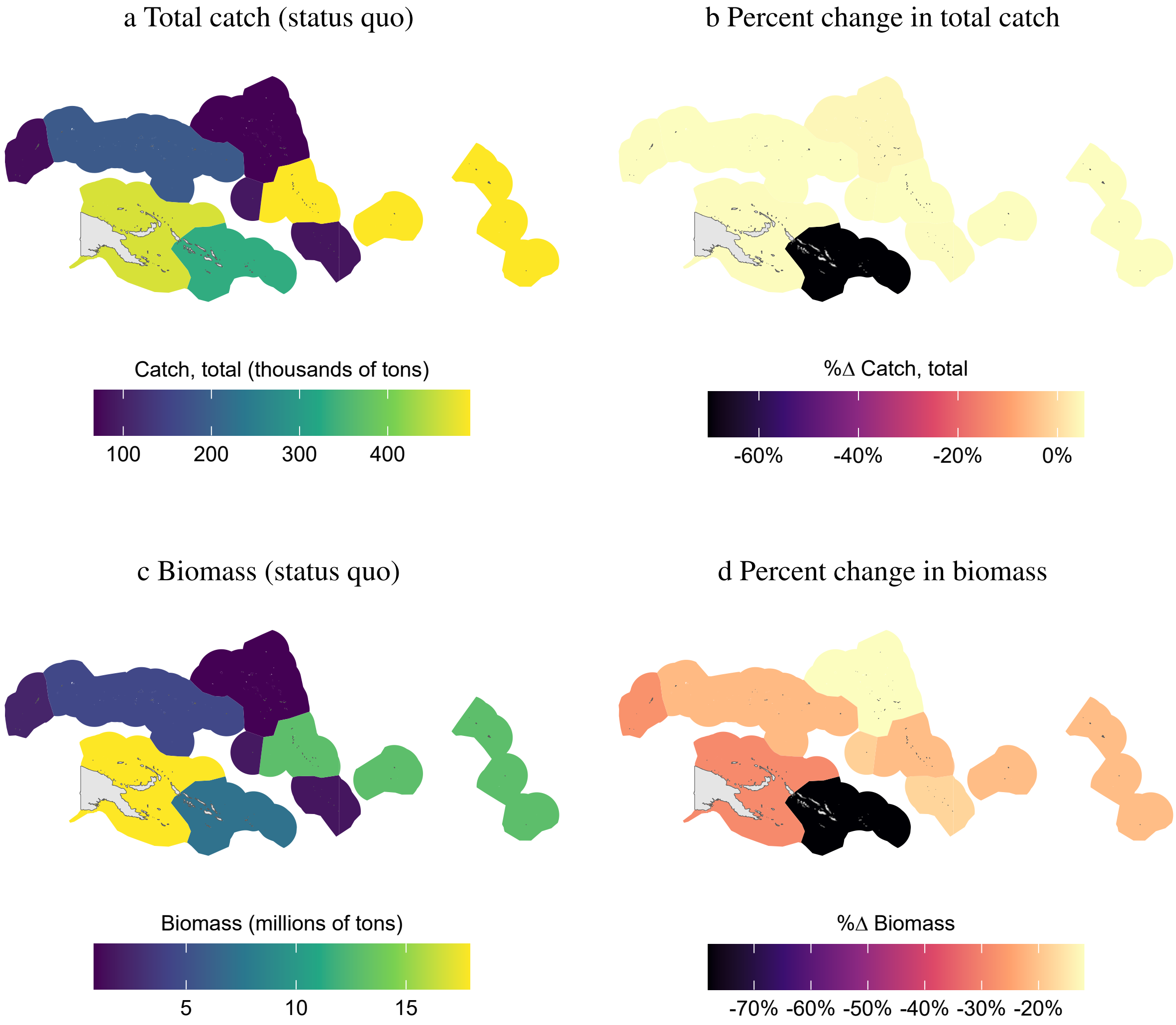

Figure S27: **Total catch and biomass if PNA did not exist when $\frac{b}{b_{MSY}} = 1.3$.** (a) Total catch (access plus non-access catch) and (c) biomass by selling country in the status quo scenario that the Parties to the Nauru Agreement (PNA) coalition exists; percent change in (b) total catch and (d) biomass if the PNA coalition did not exist. Percent change is relative to status quo value. In our baseline specification we calculate each selling country's true status quo biomass with the assumption that $\frac{b}{b_{MSY}} = 0.8$ (Methods). As a robustness check, we set $\frac{b}{b_{MSY}} = 1.3$ and then we repeat our analysis.

	Status quo	Coalition	Difference	% Difference
Catch, access (millions of tons)	2.28	1.63	-0.65	-28%
Access fee per ton	$128.20	$151.54	$23.34	18%
African sellers' profit (millions)	$150.83	$185.37	$34.54	23%
Foreign buyers' profit (millions)	$342.74	$289.81	-$52.93	-15%
Catch, non-access (millions of tons)	8.45	9.27	0.81	10%
Catch, total (millions of tons)	10.74	10.90	0.16	2%
Biomass (millions of tons)	102.70	120.50	17.81	17%

Table S1: **Effect of Africa Coalition on catch, profit, and biomass when fishing hours threshold is twice the baseline value.** Catch and profit are annual quantities. Access fee and profit are in 2020 USD. The baseline fishing hours threshold is 2,420 (Methods). As a robustness check, we use a fishing hours threshold of 4,840 to identify selling and buying countries, and then we repeat our analysis with these selling and buying countries.

	Status quo	Coalition	Difference	% Difference
Catch, access (millions of tons)	2.47	1.80	-0.66	-27%
Access fee per ton	$128.20	$149.87	$21.67	17%
African sellers' profit (millions)	$162.15	$202.22	$40.07	25%
Foreign buyers' profit (millions)	$363.15	$310.42	-$52.73	-15%
Catch, non-access (millions of tons)	9.46	10.67	1.21	13%
Catch, total (millions of tons)	11.93	12.47	0.55	5%
Biomass (millions of tons)	96.03	117.07	21.03	22%

Table S2: **Effect of Africa Coalition on catch, profit, and biomass when $\frac{b}{b_{MSY}} = 0.6$.** Catch and profit are annual quantities. Access fee and profit are in 2020 USD. In our baseline specification we calculate each selling country's true status quo biomass with the assumption that $\frac{b}{b_{MSY}} = 0.8$ (Methods). As a robustness check, we set $\frac{b}{b_{MSY}} = 0.6$ and then we repeat our analysis.

	Status quo	Coalition	Difference	% Difference
Catch, access (millions of tons)	2.47	1.93	-0.54	-22%
Access fee per ton	$128.20	$146.05	$17.85	14%
African sellers' profit (millions)	$162.15	$210.26	$48.11	30%
Foreign buyers' profit (millions)	$363.15	$323.44	-$39.71	-11%
Catch, non-access (millions of tons)	9.46	11.15	1.69	18%
Catch, total (millions of tons)	11.93	13.08	1.16	10%
Biomass (millions of tons)	77.54	102.13	24.59	32%

Table S3: **Effect of Africa Coalition on catch, profit, and biomass when $\frac{b}{b_{MSY}} = 0.4$.** Catch and profit are annual quantities. Access fee and profit are in 2020 USD. In our baseline specification we calculate each selling country's true status quo biomass with the assumption that $\frac{b}{b_{MSY}} = 0.8$ (Methods). As a robustness check, we set $\frac{b}{b_{MSY}} = 0.4$ and then we repeat our analysis.

	Status quo	Coalition	Difference	% Difference
Catch, access (millions of tons)	2.44	2.02	-0.42	-17%
Access fee per ton	$128.20	$140.95	$12.75	10%
African sellers' profit (millions)	$211.70	$236.79	$25.09	12%
Foreign buyers' profit (millions)	$368.67	$335.28	-$33.39	-9%
Catch, non-access (millions of tons)	9.49	10.04	0.56	6%
Catch, total (millions of tons)	11.93	12.06	0.13	1%
Biomass (millions of tons)	117.20	128.70	11.50	10%

Table S4: **Effect of Africa Coalition on catch, profit, and biomass when $\eta = 0.5$.** Catch and profit are annual quantities. Access fee and profit are in 2020 USD. We assume $\eta = 1$ in our baseline specification (Methods). As a robustness check, we set $\eta = 0.5$ and then we repeat our analysis.

	Status quo	Coalition	Difference	% Difference
Catch, access (millions of tons)	2.49	1.56	-0.93	-37%
Access fee per ton	$128.20	$162.03	$33.83	26%
African sellers' profit (millions)	$131.76	$176.17	$44.42	34%
Foreign buyers' profit (millions)	$359.31	$284.09	-$75.22	-21%
Catch, non-access (millions of tons)	9.44	10.57	1.13	12%
Catch, total (millions of tons)	11.93	12.12	0.20	2%
Biomass (millions of tons)	117.20	141.11	23.91	20%

Table S5: **Effect of Africa Coalition on catch, profit, and biomass when $\eta = 1.5$.** Catch and profit are annual quantities. Access fee and profit are in 2020 USD. We assume $\eta = 1$ in our baseline specification (Methods). As a robustness check, we set $\eta = 1.5$ and then we repeat our analysis.

	Status quo	Coalition	Difference	% Difference
Catch, access (millions of tons)	2.48	1.47	-1.01	-41%
Access fee per ton	$128.20	$181.45	$53.25	42%
African sellers' profit (millions)	$164.14	$222.55	$58.41	36%
Foreign buyers' profit (millions)	$722.25	$606.63	-$115.62	-16%
Catch, non-access (millions of tons)	9.44	10.65	1.21	13%
Catch, total (millions of tons)	11.93	12.13	0.20	2%
Biomass (millions of tons)	117.20	142.85	25.65	22%

Table S6: **Effect of Africa Coalition on catch, profit, and biomass when $\epsilon = 1.5$.** Catch and profit are annual quantities. Access fee and profit are in 2020 USD. We assume $\epsilon = 2$ in our baseline specification (Methods). As a robustness check, we set $\epsilon = 1.5$ and then we repeat our analysis.

	Status quo	Coalition	Difference	% Difference
Catch, access (millions of tons)	2.45	1.89	-0.57	-23%
Access fee per ton	$128.20	$142.35	$14.15	11%
African sellers' profit (millions)	$160.74	$187.91	$27.17	17%
Foreign buyers' profit (millions)	$243.02	$207.64	-$35.38	-15%
Catch, non-access (millions of tons)	9.47	10.20	0.73	8%
Catch, total (millions of tons)	11.93	12.09	0.16	1%
Biomass (millions of tons)	117.20	132.36	15.16	13%

Table S7: **Effect of Africa Coalition on catch, profit, and biomass when $\epsilon = 2.5$.** Catch and profit are annual quantities. Access fee and profit are in 2020 USD. We assume $\epsilon = 2$ in our baseline specification (Methods). As a robustness check, we set $\epsilon = 2.5$ and then we repeat our analysis.

	Status quo	Regional coalitions	Difference	% Difference
Catch, access (millions of tons)	2.47	2.37	-0.09	-4%
Access fee per ton	$128.20	$130.85	$2.65	2%
African sellers' profit (millions)	$162.15	$167.56	$5.42	3%
Foreign buyers' profit (millions)	$363.15	$356.96	-$6.19	-2%
Catch, non-access (millions of tons)	9.46	9.59	0.13	1%
Catch, total (millions of tons)	11.93	11.96	0.04	0%
Biomass (millions of tons)	117.20	120.04	2.84	2%

Table S8: **Effect of regional African coalitions on continent-level catch, profit, and biomass.** Catch and profit are annual quantities. Access fee and profit are in 2020 USD.

	Status quo	No PNA	Difference	% Difference
Catch, access (millions of tons)	1.19	0.92	-0.27	-22%
Access fee per ton	$306.75	$320.50	$13.75	4%
PNA sellers' profit (millions)	$273.18	$160.67	-$112.51	-41%
Foreign buyers' profit (millions)	$469.29	$380.50	-$88.79	-19%
Catch, non-access (millions of tons)	0.64	0.31	-0.33	-51%
Catch, total (millions of tons)	1.83	1.23	-0.60	-33%
Biomass (millions of tons)	29.95	14.44	-15.52	-52%

Table S9: **Catch, profit, and biomass if PNA did not exist.** Catch and profit are annual quantities. Access fee and profit are in 2020 USD.

	Status quo	No PNA	Difference	% Difference
Catch, access (millions of tons)	0.59	0.70	0.10	18%
Access fee per ton	$306.75	$274.76	-$31.99	-10%
PNA sellers' profit (millions)	$136.59	$103.59	-$33.00	-24%
Foreign buyers' profit (millions)	$234.64	$247.24	$12.60	5%
Catch, non-access (millions of tons)	1.06	0.83	-0.23	-22%
Catch, total (millions of tons)	1.66	1.53	-0.13	-8%
Biomass (millions of tons)	29.95	22.69	-7.26	-24%

Table S10: **Catch, profit, and biomass if PNA did not exist when access catch is half of foreign catch.** Instead of assuming access catch equals foreign catch, in this robustness check we assume access catch equals half of foreign catch in PNA country waters, and then we repeat our analysis. Catch and profit are annual quantities. Access fee and profit are in 2020 USD.

	Status quo	No PNA	Difference	% Difference
Catch, access (millions of tons)	1.19	1.25	0.07	6%
Access fee per ton	$306.75	$286.03	-$20.73	-7%
PNA sellers' profit (millions)	$273.18	$195.27	-$77.91	-29%
Foreign buyers' profit (millions)	$469.29	$462.36	-$6.92	-1%
Catch, non-access (millions of tons)	0.64	0.42	-0.22	-35%
Catch, total (millions of tons)	1.83	1.67	-0.16	-9%
Biomass (millions of tons)	49.93	33.87	-16.06	-32%

Table S11: **Catch, profit, and biomass if PNA did not exist when $\frac{b}{b_{MSY}} = 1.3$.** In our baseline specification we calculate each selling country's true status quo biomass with the assumption that $\frac{b}{b_{MSY}} = 0.8$ (Methods). As a robustness check, we set $\frac{b}{b_{MSY}} = 1.3$ and then we repeat our analysis. Catch and profit are annual quantities. Access fee and profit are in 2020 USD.

Supplementary derivations

In Hendricks and McAfee (2010), the market mechanism solves for the quantity that equates the marginal opportunity cost with the marginal fishing profit, both of which are evaluated at the market-level values. Re-writing their Equation 8 in the language of our model, and letting $'$ indicate the partial derivative operator, we have

$$c'(\frac{Q(\hat{B},\hat{T})}{\hat{B}}) = v'(\frac{Q(\hat{B},\hat{T})}{\hat{T}}) \tag{11}$$

In their constant elasticities special case, the marginal opportunity cost and marginal fishing profit take the forms

$$(\frac{Q(\hat{B},\hat{T})}{\hat{B}})^{1/\eta} = (\frac{Q(\hat{B},\hat{T})}{\hat{T}})^{-1/\epsilon} \tag{12}$$

(bottom right column of page 397). We can re-arrange Equation 12 to solve for the equilibrium quantity, which we stated in Equation 4:

$$\begin{aligned}
\frac{Q(\hat{B},\hat{T})}{\hat{B}} &= (\frac{Q(\hat{B},\hat{T})}{\hat{T}})^{-\eta/\epsilon} \\
(\frac{Q(\hat{B},\hat{T})}{\hat{B}})^{\epsilon} &= (\frac{Q(\hat{B},\hat{T})}{\hat{T}})^{-\eta} \\
(\frac{Q(\hat{B},\hat{T})}{\hat{B}})^{\epsilon} &= (\frac{\hat{T}}{Q(\hat{B},\hat{T})})^{\eta} \\
Q(\hat{B},\hat{T})^{\epsilon+\eta} &= \hat{B}^{\epsilon}\hat{T}^{\eta} \Rightarrow \\
Q(\hat{B},\hat{T}) &= \hat{B}^{\epsilon/(\epsilon+\eta)}\hat{T}^{\eta/(\epsilon+\eta)}
\end{aligned} \tag{13}$$

The equilibrium price, which we stated in Equation 3, occurs when the marginal opportunity cost equals the marginal fishing price for all sellers and buyers. Re-writing their Equation 5 in

the language of our model, we have

$$c'(\frac{q_i}{\hat{b}_i}) = p = v'(\frac{q_j}{\hat{t}_j}) \ \forall i,j \tag{14}$$

We solve for the equilibrium price in the constant elasticities case by re-arranging the left side of Equation 14, substituting $\frac{\hat{b}_i Q(\hat{B},\hat{T})}{\hat{B}}$ for q_i, and plugging in the equilibrium quantity:

$$\begin{aligned}
c'(\frac{\hat{b}_i Q(\hat{B},\hat{T})}{\hat{B}\hat{b}_i}) &= p(\hat{B},\hat{T}) \\
c'(\frac{Q(\hat{B},\hat{T})}{\hat{B}}) &= p(\hat{B},\hat{T}) \\
(\frac{Q(\hat{B},\hat{T})}{\hat{B}})^{1/\eta} &= p(\hat{B},\hat{T}) \\
(\frac{\hat{B}^{\epsilon/(\epsilon+\eta)}\hat{T}^{\eta/(\epsilon+\eta)}}{\hat{B}})^{1/\eta} &= p(\hat{B},\hat{T}) \\
(\frac{\hat{T}^{\eta/(\epsilon+\eta)}}{\hat{B}^{\eta/(\epsilon+\eta)}})^{1/\eta} &= p(\hat{B},\hat{T}) \Rightarrow \\
p(\hat{B},\hat{T}) &= \hat{B}^{-1/(\epsilon+\eta)}\hat{T}^{1/(\epsilon+\eta)}
\end{aligned} \tag{15}$$

Supplementary bibliography

Hendricks, K. & McAfee, R. P. A theory of bilateral oligopoly. *Economic Inquiry* **48** (2), 391–414 (2010).
