# Supplementary material for: A fish cartel for Africa
Source: Nat Commun. 2023 Nov 13;14:7124. doi: 10.1038/s41467-023-42886-z (PMC10643414; doi:10.1038/s41467-023-42886-z)
Supplement: Supplementary file 3 — Reporting Summary [file 41467_2023_42886_MOESM3_ESM.pdf]

nature portfolio

Corresponding author(s): Gabriel Englander

Last updated by author(s): October 7, 2023

# Reporting Summary

Nature Portfolio wishes to improve the reproducibility of the work that we publish. This form provides structure for consistency and transparency in reporting. For further information on Nature Portfolio policies, see our Editorial Policies and the Editorial Policy Checklist.

## Statistics

For all statistical analyses, confirm that the following items are present in the figure legend, table legend, main text, or Methods section.

| n/a | Confirmed | |
|---|---|---|
| ☐ | ☒ | The exact sample size (*n*) for each experimental group/condition, given as a discrete number and unit of measurement |
| ☒ | ☐ | A statement on whether measurements were taken from distinct samples or whether the same sample was measured repeatedly |
| ☒ | ☐ | The statistical test(s) used AND whether they are one- or two-sided<br>*Only common tests should be described solely by name; describe more complex techniques in the Methods section.* |
| ☒ | ☐ | A description of all covariates tested |
| ☐ | ☒ | A description of any assumptions or corrections, such as tests of normality and adjustment for multiple comparisons |
| ☐ | ☒ | A full description of the statistical parameters including central tendency (e.g. means) or other basic estimates (e.g. regression coefficient) AND variation (e.g. standard deviation) or associated estimates of uncertainty (e.g. confidence intervals) |
| ☒ | ☐ | For null hypothesis testing, the test statistic (e.g. *F*, *t*, *r*) with confidence intervals, effect sizes, degrees of freedom and *P* value noted<br>*Give P values as exact values whenever suitable.* |
| ☒ | ☐ | For Bayesian analysis, information on the choice of priors and Markov chain Monte Carlo settings |
| ☒ | ☐ | For hierarchical and complex designs, identification of the appropriate level for tests and full reporting of outcomes |
| ☒ | ☐ | Estimates of effect sizes (e.g. Cohen's *d*, Pearson's *r*), indicating how they were calculated |

*Our web collection on statistics for biologists contains articles on many of the points above.*

## Software and code

Policy information about availability of computer code

| | |
|---|---|
| Data collection | Most data were collected without any code; they were manually recorded in .csv files using Google Sheets, or they were downloaded manually from the websites of data providers. Other data were downloaded using R version 4.1.0. All data and code are available in figshare at https://doi.org/10.6084/m9.figshare.23948991. |
| Data analysis | All data were analyzed using R version 4.1.0. |

For manuscripts utilizing custom algorithms or software that are central to the research but not yet described in published literature, software must be made available to editors and reviewers. We strongly encourage code deposition in a community repository (e.g. GitHub). See the Nature Portfolio guidelines for submitting code & software for further information.

## Data

Policy information about availability of data

All manuscripts must include a data availability statement. This statement should provide the following information, where applicable:

- Accession codes, unique identifiers, or web links for publicly available datasets
- A description of any restrictions on data availability
- For clinical datasets or third party data, please ensure that the statement adheres to our policy

All data are available in figshare at https://doi.org/10.6084/m9.figshare.23948991. We include this DOI in the Data Availability Statement. We also cite the dataset in the manuscript.

## Human research participants

Policy information about studies involving human research participants and Sex and Gender in Research.

| | |
|---|---|
| Reporting on sex and gender | NA |
| Population characteristics | NA |
| Recruitment | NA |
| Ethics oversight | NA - not human subjects research |

Note that full information on the approval of the study protocol must also be provided in the manuscript.

# Field-specific reporting

Please select the one below that is the best fit for your research. If you are not sure, read the appropriate sections before making your selection.

☐ Life sciences ☒ Behavioural & social sciences ☐ Ecological, evolutionary & environmental sciences

For a reference copy of the document with all sections, see nature.com/documents/nr-reporting-summary-flat.pdf

# Behavioural & social sciences study design

All studies must disclose on these points even when the disclosure is negative.

| | |
|---|---|
| Study description | All data are quantitative. Study develops a theoretical model, parameterizes the model with data, and uses the model to simulate different scenarios. |
| Research sample | Primary data are African and Pacific Island countries' fishing access agreements. Study includes all of these data between 2010 and 2021 that are publicly available. Study also uses all fishing hours and fishing vessels data in these countries' waters between 2016 and 2020 from Global Fishing Watch; all catch data in these countries' waters between 2010 and 2018 from the Sea Around Us; and biological parameters of all stocks in these countries' waters from Costello et al. (2016; PNAS). |
| Sampling strategy | No sampling or sample size calculation performed. We used the universe of relevant and available data for the waters of African and Pacific Island countries. |
| Data collection | Fishing access agreement data recorded manually in .csv files using Google Sheets. Other data downloaded manually from the websites of data providers, or else using R version 4.1.0. |
| Timing | Data collected between September 2021 and June 2022. |
| Data exclusions | We identify buying and selling countries as follows. For the African Access Market, we set the fishing hours threshold as the maximum value that would include all selling countries for whom we observe an access agreement between 2016 and 2020 as participants in the African Access Market. In other words, we choose the largest fishing hours threshold that would not exclude any selling country that we know sells access to its waters. This rule yields 32 selling countries. We use the same threshold to identify 33 buying countries; in at least one year, all of these countries fish at least as many hours as the threshold in at least one selling country. The threshold is 2,420 hours. In the Pacific Island countries analysis, we use this same fishing hours threshold to identify buying countries. However, we do not need to identify selling countries for this analysis because the identities of the nine Parties to the Nauru Agreement are public knowledge. |
| Non-participation | NA |
| Randomization | NA |

# Reporting for specific materials, systems and methods

We require information from authors about some types of materials, experimental systems and methods used in many studies. Here, indicate whether each material, system or method listed is relevant to your study. If you are not sure if a list item applies to your research, read the appropriate section before selecting a response.

## Materials & experimental systems

| n/a | Involved in the study |
| --- | --- |
| ☒ | ☐ Antibodies |
| ☒ | ☐ Eukaryotic cell lines |
| ☒ | ☐ Palaeontology and archaeology |
| ☒ | ☐ Animals and other organisms |
| ☒ | ☐ Clinical data |
| ☒ | ☐ Dual use research of concern |

## Methods

| n/a | Involved in the study |
| --- | --- |
| ☒ | ☐ ChIP-seq |
| ☒ | ☐ Flow cytometry |
| ☒ | ☐ MRI-based neuroimaging |
